# What do primary care staff know and do about blood borne virus testing and care for migrant patients? A national survey

**DOI:** 10.1186/s12889-020-10068-x

**Published:** 2021-02-11

**Authors:** Rachel Roche, Ruth Simmons, Alison F. Crawshaw, Pip Fisher, Manish Pareek, Will Morton, Theresa Shryane, Kristina Poole, Arpana Verma, Ines Campos-Matos, Sema Mandal

**Affiliations:** 1 Blood Safety, Hepatitis, Sexually Transmitted Infections (STI) and HIV Service, National Infection Service, Public Health England, 61 Colindale Avenue, London, NW9 5EQ UK; 2 The National Institute for Health Research Health Protection Research Unit (NIHR HPRU) in Blood Borne and Sexually Transmitted Infections at University College London, Gower St, London, WC1E 6BT UK; 3 Migration Health, Health Improvement, Public Health England, Wellington House, 133-155 Waterloo Road, London, SE1 8UG UK; 4 Institute for Infection and Immunity, St George’s, University of London, Cranmer Terrace, Tooting, London, SW17 0RE UK; 5 University of Manchester, Oxford Road, Manchester, M13 9PL UK; 6 Department of Respiratory Sciences, University of Leicester, University Road, Leicester, LE1 7RH UK; 7 Public Health England Yorkshire and the Humber, 2nd Floor, Blenheim House, Duncombe Street, Leeds, LS1 4PL UK; 8 Public Health England North West, 2nd Floor, 3 Piccadilly Place, London Road, Manchester, M1 3BN UK; 9 Institute of Epidemiology and Healthcare, University College London (UCL), 1-19 Torrington Place WC1E 7HB, London, UK

**Keywords:** Hepatitis B, Hepatitis C, HIV, Blood borne virus, Testing, Screening, General practice, Migrants, Healthcare access, UK

## Abstract

**Background:**

UK migrants born in intermediate to high prevalence areas for blood borne viruses (BBV) including hepatitis B, hepatitis C and HIV are at increased risk of these infections. National guidance from Public Health England (PHE) and National Institute for Health and Care Excellence (NICE) recommends primary care test this population to increase diagnoses and treatment. We aimed to investigate primary care professionals’ knowledge of entitlements, and perceptions of barriers, for migrants accessing healthcare, and their policies, and reported practices and influences on provision of BBV testing in migrants.

**Methods:**

A pre-piloted questionnaire was distributed between October 2017 and January 2018 to primary care professionals attending the Royal College of General Practitioners and Best Practice in Primary Care conferences, via a link in PHE Vaccine Updates and through professional networks.

Survey results were analysed to give descriptive statistics, and responses by respondent characteristics: profession, region, practice size, and frequency of seeing migrant patients. Responses were considered on a per question basis with response rates for each question presented with the results.

**Results:**

Four hundred fourteen questionnaires were returned with responses varying by question, representing an estimated 5.7% of English GP practices overall. Only 14% of respondents’ practices systematically identified migrant patients for testing. Universal opt-out testing was offered to newly registering migrant patients by 18% of respondents for hepatitis B, 17% for hepatitis C and 21% for HIV. Knowledge of healthcare entitlements varied; fewer clinical staff knew that general practice consultations were free to all migrants (76%) than for urgent care (88%). Performance payment structure (76%) had the greatest reported influence on testing, followed by PHE and Clinical Commissioning Group recommendations (73% each). Language and culture were perceived to be the biggest barriers to accessing care.

**Conclusions:**

BBV testing for migrant patients in primary care is usually ad hoc, which is likely to lead to testing opportunities being missed. Knowledge of migrants’ entitlements to healthcare varies and could affect access to care. Interventions to improve professional awareness and identification of migrant patients requiring BBV testing are needed to reduce the undiagnosed and untreated burden of BBVs in this vulnerable population.

## Background

Migrants in the UK from intermediate or high prevalence areas for HIV, hepatitis B (HBV) and hepatitis C (HCV) are at increased risk of these infections compared to the UK-born population, and experience a disproportionate burden of disease [1–3]. In 2017 almost half (49%) of newly diagnosed cases of HIV among heterosexuals were among black Africans or heterosexuals born in a high prevalence country [2] and an estimated 95% of new chronic HBV infections in the UK are among individuals who probably acquired their infection overseas in endemic countries, often perinatally or in childhood [4]. For all three blood borne viruses (BBV) the undiagnosed fraction remains high, with a high proportion of infections diagnosed late, potentially contributing to avoidable morbidity and mortality and increased risk of onward transmission [5–8].

In 2016, the UK government signed up to the World Health Organisation (WHO) goal of eliminating viral hepatitis as a public health threat by 2030 [9]. HBV and HCV testing in primary care for migrants from countries with intermediate or high prevalence (> 2%) has been recommended by National Institute for Health and Care Excellence (NICE) guidance since 2012 [10], and since 2018 European Centre for Disease Prevention and Control (ECDC) recommends screening newly arrived migrants for BBV [11]. Although surveillance data indicates that an increasing proportion of BBV diagnoses take place in primary care, evidence suggests a low proportion of eligible migrants receive the recommended BBV testing in this setting [12, 13].

Language, stigma and poor knowledge and understanding of disease create barriers for migrants to accessing testing and treatment for viral hepatitis [14, 15] and migrants often experience discrimination and bureaucratic obstacles when trying to access healthcare [16, 17]. In August 2017 UK healthcare entitlements for migrants changed and the list of chargeable services was expanded [18, 19]. While many services, including primary care, are still free to all migrants, for others a patient should now be deemed ordinarily resident in the UK to access services for free. Sexual health, family planning, GP and nurse consultations in primary care, emergency department (ED) and walk-in centres, and treatment of mental and physical conditions caused by torture, female genital mutilation (FGM), domestic or sexual violence are free to all migrants, while communicable disease diagnosis and treatment is free to all for a list of specified diseases, including HIV, viral hepatitis, TB and Middle East respiratory syndrome [18–20]. For surgery or outpatient services in secondary care, a patient should be deemed ordinarily resident in the UK to access the service free of charge. This additional complexity may lead to uncertainty among both migrants and professionals about entitlements, and could create additional barriers to accessing services.

We, therefore, conducted a national survey to understand primary care staff’s knowledge, attitudes, policies and practice regarding BBVs in migrant patients, influences on these, and their perceptions of the barriers for migrants in accessing healthcare.

## Methods

### Study design

A cross-sectional survey conducted among primary care staff in England.

#### Study population

The target population for this study were primary care staff working in general practices in England. All primary care staff were eligible for inclusion, but it was expected that respondents would primarily be general practitioners (GPs) and practice nurses.

#### Sampling procedures

A convenience sampling approach was used, with survey participants recruited through several routes during October 2017. Firstly, the link to the online survey was circulated via primary care networks of local Public Health England (PHE) Health Protection Teams across England. Secondly, the link to the online survey was circulated in the PHE publication Vaccine Updates, a newsletter primarily subscribed to by general practice professionals with distribution ~ 46,000. These two routes were used opportunistically and in parallel in order to access primary care professionals, as we did not have access to another primary care network with equivalent coverage across England.

In addition, stalls were held at the Royal College of General Practitioners (RCGP) annual conference in Liverpool and the Best Practice in Primary Care conference in Birmingham, both in October 2017. Attendees were approached and asked to participate in the survey via tablet devices, or offered shorter paper versions of the survey to take away and return once completed.

Due to the timeframe and budget of the project, participants were not followed up to respond to the survey, and the online survey was closed in January 2018.

#### Data collection

The survey was created as an online questionnaire using SelectSurvey.net™ software (ClassApps LLC, Kansas City, Missouri) and responses were exported into Excel for analysis. Shorter paper versions of the survey were also distributed among participants attending the two primary care conferences. Responses from paper surveys were manually input into Excel by the lead author.

### Questionnaire design

The questionnaire was developed at PHE in collaboration with a project group of clinicians, academic GPs and qualitative researchers and piloted before launch with a group of 4 academic GPs.

The questionnaire consisted of 37 questions covering the following topics.

1. Respondent and practice characteristics
2. Clinical questions analysed for clinical staff (GPs and nurses) only: Knowledge of migrants’ entitlements to health services; Issues considered for newly registering migrant patients; Knowledge of testing serology; Management of patients diagnosed with hepatitis B and C; Incentives / motivation for BBV testing
3. Practice policy questions analysed for all respondents: Practice policy for offering BBV testing to migrants; Identification of migrants for BBV testing; Recording of routine data
4. Barriers questions analysed for all respondents: Perceived barriers for migrants to accessing healthcare across the care pathway: Healthcare; BBV testing in primary care; Secondary care for patients with BBV; Barriers for asylum seekers

The full questionnaire took approximately 17 min to complete (see additional file 1 for full online questionnaire and additional file 2 for shorter paper questionnaire). Most questions were multiple choice, apart from those relating to barriers, practice size and practice population which were free text.

To investigate knowledge of healthcare entitlements respondents were asked, as of August 2017, which services were free irrespective of migration status, using legislation and implementation guidelines to define correct responses [18, 19].

Respondents were asked about location of practice and professional role. No additional respondent characteristics were sought as the focus of the survey was on practice, not individual characteristics.

### Definitions

A migrant is defined as someone who changes their country of usual residence, in whatever manner, for whatever reason and irrespective of their legal status. The UN defines a permanent migrant as someone who moves to a country for a period of 12 or more months, often for work, education or joining family, effectively making it their new country of usual residence [21]. A refugee is defined as someone who has fled their home country for reasons of feared persecution, conflict, generalised violence or other circumstances that have seriously disturbed public order and are seeking protection in another country [21]. In the UK, for a person to be officially recognised as a refugee they first have to make a claim for asylum and have this accepted by the government. An asylum seeker is a person who seeks safety from persecution or serious harm in a country other than their own and has formally applied for asylum (i.e. refugee status) from that country, but is awaiting a decision on their application [22].

A definitions sheet was provided with the survey to help respondents understand terms relating to migration which also included refused asylum seeker and undocumented migrant (not specifically mentioned in the questionnaire) (additional file 3).

Definitions of other terms used are as follows: Clinical staff: respondents who identified their role as either GP (GP partner, Salaried GP, Locum GP, or ‘other’ and specified GP), or nurse (Practice Nurse or ‘other’ and specified nurse). In questions relating to practice policy for BBV testing, new migrants refer to newly registering patients who were migrants from outside the UK (regardless of when they had migrated), and existing migrants refer to patients already registered with the practice who had ever migrated from outside the UK. When asking about practice characteristics, the proportion of new migrants on a practice list was defined as migrants registered with the practice who had lived in the UK for 5 years or less, regardless of when they registered.

The roles of GPs and nurses in the diagnosis and management of BBVs vary by practice. Practice nurses undertake a range of nursing assessments and provide appropriate care / treatment in conjunction with GPs according to practice policy and protocols and are likely to be responsible for vaccination and testing, whereas GPs would usually be responsible for clinical diagnoses and referrals to secondary care.

Practice size was defined as small, medium or large using tertiles of practice populations in England reported by NHS Digital for December 2017 [23].

### Data analysis

Results were collated and de-duplicated using email addresses. Practice names were checked to identify if there were multiple respondents from the same practice, but these were not de-duplicated as responses differed and were from different professionals.

Due to the methods used to circulate the survey via an e-bulletin to which practices subscribe, it was not possible to calculate a formal response rate. Instead, the number of responses as a proportion of GP practices in England (based on NHS data from 2017) was calculated to provide an indication of the proportion of England GP practices represented in responses [23].

Results were analysed in Excel and R for overall descriptive statistics (percentages and 95% confidence intervals (CI)) and then stratified by respondent and practice characteristics. Responses for clinical knowledge and practice questions were restricted to those who gave their role as GP or nurse and stratified by profession. Responses for practice policy questions were analysed for all respondents, and stratified by region, frequency of seeing migrant patients, and practice size.

To assess the statistical significance of differences in responses to questions, and for differences by stratified analysis, variables were dichotomised where required before performing Chi squared tests. Where this was done, the dichotomous outcome is highlighted in bold in results tables. Fishers exact tests were used where the cross-tabulation did not meet the criteria for Chi squared tests. Differences were considered statistically significant where *p* < 0.05.

Free text responses on barriers were grouped by thematic analysis into 10 themes: language/culture, patient information/knowledge, staff information/knowledge, psychological, accommodation, prejudice/discrimination, patient financial, organisational resource, geographical, and service/organisational issues.

Each question had a different response rate, and descriptive statistics were based on the number of respondents for each question, which are presented with the results.

## Results

### Respondent and practice characteristics

The survey was circulated through the PHE Vaccine Updates circulation list, although it is not known how many will have actively read this, and others may have been forwarded the survey link by contacts. After deduplication 414 responses were obtained; 16 paper and 398 online, an estimated 5.7% response rate, based on the number of GP practices in England. The majority of responses came from circulation of the survey link as response at conferences was poor; no attendees completed the online survey on the day, and only 16 paper surveys were returned. Where practice name was reported (145 respondents), there were 3 practices that each had 2 respondents, all other practices were unique. Most respondents were practice nurses (49%, 202/414) and GPs (40%, 165/414) with the highest responses from the Midlands (21%, 86/414), the South East (16%, 65/414) and London (15%, 63/414) (Table 1). Responses were equally distributed among those who reported they saw migrant patients frequently (34%, 108/317), sometimes (33%, 103/317) and rarely (33%, 106/317) (77% response rate).

**Table 1:** Respondent and practice characteristics

		Overall	
		Number (%)	95% CI
**1a: Respondent characteristics**			
**Position**	General Practitioner (GP)	165 (39.9)	35.3–44.6
	Nurse	202 (48.8)	44.0–53.6
	Practice Manager	29 (7.0)	4.9–9.9
	Other clinical	6 (1.4)	0.7–3.1
	Other non-clinical	7 (1.7)	0.8–3.4
	Not stated	5 (1.2)	0.5–2.8
	Total responses	414	
**Frequency of seeing migrant patients**	Frequently	108 (34.1)	29.1–39.4
	Sometimes	103 (32.5)	27.6–37.8
	Rarely	106 (33.4)	28.5–38.8
	Total responses	317	
**1b: Practice characteristics**			
**Location**	East of England	36 (8.7)	6.3–11.8
	London	63 (15.2)	12.1–19.0
	Midlands	86 (20.8)	17.1–24.9
	North East	23 (5.6)	3.7–8.2
	North West	60 (14.5)	11.4–18.2
	South East	65 (15.7)	12.5–19.5
	South West	30 (7.2)	5.1–10.2
	Yorkshire & the Humber	51 (12.3)	9.5–15.8
	Total responses	414	
**Size of practice**	Small (0–5279 patients)	39 (25.8)	19.5–33.3
	Medium (5280–9299 patients)	42 (27.8)	21.3–35.4
	Large (> = 9300 patients)	70 (46.4)	38.6–54.3
	Total responses	151	
**Proportion of practice population that are new migrants**	Low (0–9%)	40 (50.6)	39.8–61.4
	Medium (10–24% patients)	25 (31.6)	22.4–42.5
	High (25–49% patients)	4 (5.1)	2.0–12.3
	Very high (50–100% patients)	10 (12.7)	7.0–21.8
	Total responses	79	
**Proportion of practice population that are asylum seekers/refugees**	Low (0–9%)	57 (76.0)	65.2–84.2
	Medium (10–24% patients)	7 (9.3)	4.6–18.0
	High (25–49% patients)	3 (4.0)	1.4–11.1
	Very high (50–100% patients)	8 (10.7)	5.5–19.7
	Total responses	75	

Practice characteristics were poorly completed; only 151 (38%) respondents reported their practice size and of these 26% (39/151) were small (< 5280 patients), 28% (42/151) were medium (5280–9300 patients) and 46% (70/151) were large (> = 9300 patients). Of the 79 (20%) respondents who reported the proportion of new migrants (< 5 years in the UK) on their practice list, 50% (40/79) had < 10, 32% (25/79) had 10–24% and 13% (10/79) had over 50%. Similarly, of the 75 (19%) who reported the proportion of their practice list that were asylum seekers/refugees, 76% (57/75) had < 10, 9% (7/75) had 10–24% and only 11% (8/75) had over 50%. There were no significant differences in practice or other respondent characteristics by professional role (*p* > 0.2).

## Professional knowledge and practice

### Issues considered for newly registering patients who are migrants

When asked which issues were always addressed for newly registering patients who are migrants (response rate 78% of GPs and nurses), vaccination history was the most common always addressed issue (58.2%, 167/287, 95% CI 52.4–63.7), and family planning, TB screening, sexual health and BBV screening more often considered on a situation-specific basis (Table 2). The proportion that never addressed issues ranged from 4.9% (14/287, 2.9–8.0) for vaccination history and family planning to 11.8% (34/287, 8.6–16.1) for TB screening. BBV risk assessments were considered for all newly registering migrant patients by 29.6% (85/287, 24.6–35.1), 27.2% (78/287, 22.4–32.6) and 30.0% (86/287, 25.0–35.5) for HBV, HCV and HIV respectively and this did not differ by the respondent’s profession (*p* = 0.763, *p* = 0.953 and *p* = 0.867 respectively). TB screening was addressed for all newly registering migrant patients by 28.6% (82/287, 23.7–34.1) of respondents, and this did not differ by profession (*p* = 0.072). There were significant differences in practice by profession for vaccination history, family planning, and sexual health advice and screening (all *p* < 0.01), with nurses more likely than GPs to address these issues for all newly registering migrant patients.

**Table 2:** Primary care clinical staff knowledge, perceptions and practice regarding BBV testing and care for migrant patients

Question			Overall - GPs and Nurses Number (%, 95CI)	GPs Number (%, 95CI)	Nurses Number (%, 95CI)	P (GPs vs nurses)
**Which issues do you address when speaking to new migrant patients?**	**Vaccination history**	**All new migrants**	**167 (58.2,** **52.4–63.7)**	**58 (41.7,** **33.9–50.0)**	**109 (73.6,** **66.0–80.1)**	< 0.0001
		Asylum seekers / refugees only	9 (3.1, 1.7–5.9)	4 (2.9, 1.1–7.2)	5 (3.4, 1.5–7.7)	
		Never considered	14 (4.9, 2.9–8.0)	12 (8.6, 5.0–14.5)	2 (1.4, 0.4–4.8)	
		Situation specific	97 (33.8, 28.6–39.5)	65 (46.8, 38.7–55.0)	32 (21.6, 15.8–28.9)	
		Total	287	139	148	
	**HIV risk assessment**	**All new migrants**	**86 (30.0, 25.0–35.5)**	**41 (29.5, 22.5–37.5)**	**45 (30.4, 23.6–38.2)**	0.867
		Asylum seekers / refugees only	7 (2.4, 1.2–4.9)	0 (0.0, 0.0–2.7)	7 (4.7, 2.3–9.4)	
		Never considered	23 (8.0, 5.4–11.7)	8 (5.8, 2.9–10.9)	15 (10.1, 6.2–16.0)	
		Situation specific	171 (59.6, 53.8–65.1)	90 (64.7, 56.5–72.2)	81 (54.7, 46.7–62.5)	
		Total	287	139	148	
	**Hepatitis B risk assessment**	**All new migrants**	**85 (29.6, 24.6–35.1)**	**40 (28.8, 21.9–36.8)**	**45 (30.4, 23.6–38.2)**	0.763
		Asylum seekers / refugees only	11 (3.8, 2.2–6.7)	1 (0.7, 0.1–4.0)	10 (6.8, 3.7–12.0)	
		Never considered	24 (8.4, 5.7–12.1)	10 (7.2, 4.0–12.7)	14 (9.5, 5.7–15.3)	
		Situation specific	167 (58.2, 52.4–63.7)	88 (63.3, 55.0–70.9)	79 (53.4, 45.4–61.2)	
		Total	287	139	148	
	**Hepatitis C risk assessment**	**All new migrants**	**78 (27.2, 22.4–32.6)**	**38 (27.3, 20.6–35.3)**	**40 (27.0, 20.5–34.7)**	0.953
		Asylum seekers / refugees only	12 (4.2, 2.4–7.2)	1 (0.7, 0.1–4.0)	11 (7.4, 4.2–12.8)	
		Never considered	29 (10.1, 7.1–14.1)	10 (7.2, 4.0–12.7)	19 (12.8, 8.4–19.2)	
		Situation specific	168 (58.5, 52.8–64.1)	90 (64.7, 56.5–72.2)	78 (52.7, 44.7–60.6)	
		Total	287	139	148	
	**TB screening**	**All new migrants**	**82 (28.6, 23.7–34.1)**	**39 (28.1, 21.3–36.0)**	**43 (29.1, 22.3–36.8)**	0.852
		Asylum seekers / refugees only	13 (4.5, 2.7–7.6)	4 (2.9, 1.1–7.2)	9 (6.1, 3.2–11.2)	
		Never considered	34 (11.8, 8.6–16.1)	11 (7.9, 4.5–13.6)	23 (15.5, 10.6–22.2)	
		Situation specific	158 (55.1, 49.3–60.7)	85 (61.2, 52.9–68.8)	73 (49.3, 41.4–57.3)	
		Total	287	139	148	
	**Sexual health advice and screening**	**All new migrants**	**80 (27.9, 23.0–33.3)**	**27 (19.4, 13.7–26.8)**	**53 (35.8, 28.5–43.8)**	0.002
		Asylum seekers / refugees only	7 (2.4, 1.2–4.9)	0 (0.0, 0.0–2.7)	7 (4.7, 2.3–9.4)	
		Never considered	18 (6.3, 4.0–9.7)	9 (6.5, 3.4–11.8)	9 (6.1, 3.2–11.2)	
		Situation specific	182 (63.4, 57.7–68.8)	103 (74.1, 66.2–80.7)	79 (53.4, 45.4–61.2)	
		Total	287	139	148	
	**Family planning**	**All new migrants**	**86 (30.0, 25.0–35.5)**	**29 (20.9, 14.9–28.4)**	**57 (38.5, 31.1–46.5)**	0.001
		Asylum seekers / refugees only	7 (2.4, 1.2–4.9)	0 (0.0, 0.0–2.7)	7 (4.7, 2.3–9.4)	
		Never considered	14 (4.9, 2.9–8.0)	7 (5.0, 2.5–10.0)	7 (4.7, 2.3–9.4)	
		Situation specific	180 (62.7, 57.0–68.1)	103 (74.1, 66.2–80.7)	77 (52.0, 44.0–59.9)	
		Total	287	139	148	
**To the best of your knowledge, as of August 2017 which of the following services are free to all irrespective of migration status?**	**GP or nurse consultations in primary care**	**Yes**	**279 (76.0, 71.4–80.1)**	**130 (78.8, 71.9–84.3)**	**149 (73.8, 67.3–79.3)**	0.262
		No	56 (15.3, 11.9–19.3)	23 (13.9, 9.5–20.0)	33 (16.3, 11.9–22.1)	
		Don’t know	32 (8.7, 6.2–12.0)	12 (7.3, 4.2–12.3)	20 (9.9, 6.5–14.8)	
		Total	367	165	202	
	**Operations or outpatient services in secondary care**	Yes	100 (27.2, 22.9–32.0)	36 (21.8, 16.2–28.7)	64 (31.7, 25.7–38.4)	0.014
		**No**	**192 (52.3, 47.2–57.4)**	**98 (59.4, 51.8–66.6)**	**94 (46.5, 39.8–53.4)**	
		Don’t know	75 (20.4, 16.6–24.9)	31 (18.8, 13.6–25.4)	44 (21.8, 16.6–28.0)	
		Total	367	165	202	
	**Family planning**	**Yes**	**259 (70.6, 65.7–75.0)**	**115 (69.7, 62.3–76.2)**	**144 (71.3, 64.7–77.1)**	0.739
		No	32 (8.7, 6.2–12.0)	13 (7.9, 4.7–13.0)	19 (9.4, 6.1–14.2)	
		Don’t know	76 (20.7, 16.9–25.1)	37 (22.4, 16.7–29.4)	39 (19.3, 14.5–25.3)	
		Total	367	165	202	
	**Hepatitis B and C testing, diagnosis and management**	**Yes**	**268 (73.0, 68.3–77.3)**	**120 (72.7, 65.5–78.9)**	**148 (73.3, 66.8–78.9)**	0.908
		No	18 (4.9, 3.1–7.6)	8 (4.8, 2.5–9.3)	10 (5.0, 2.7–8.9)	
		Don’t know	81 (22.1, 18.1–26.6)	37 (22.4, 16.7–29.4)	44 (21.8, 16.6–28.0)	
		Total	367	165	202	
	**Treatment of mental and physical conditions caused by torture, FGM, domestic or sexual violence**	**Yes**	**299 (81.5, 77.2–85.1)**	**125 (75.8, 68.7–81.7)**	**174 (86.1, 80.7–90.2)**	0.011
		No	14 (3.8, 2.3–6.3)	12 (7.3, 4.2–12.3)	2 (1.0, 0.3–3.5)	
		Don’t know	54 (14.7, 11.5–18.7)	28 (17.0, 12.0–23.4)	26 (12.9, 8.9–18.2)	
		Total	367	165	202	
	**Communicable disease services**	**Yes**	**305 (83.3, 79.2–86.8)**	**138 (83.6, 77.2–88.5)**	**167 (83.1, 77.3–87.6)**	0.806
		No	8 (2.2, 1.1–4.3)	4 (2.4, 0.9–6.1)	4 (2.0, 0.8–5.0)	
		Don’t know	53 (14.5, 11.2–18.5)	23 (13.9, 9.5–20.0)	30 (14.9, 10.7–20.5)	
		Total	366	165	201	
	**Sexually transmitted disease services**	**Yes**	**305 (83.3, 79.2–86.8)**	**135 (81.8, 75.2–87.0)**	**170 (84.6, 78.9–88.9)**	0.552
		No	9 (2.5, 1.3–4.6)	5 (3.0, 1.3–6.9)	4 (2.0, 0.8–5.0)	
		Don’t know	52 (14.2, 11.0–18.2)	25 (15.2, 10.5–21.4)	27 (13.4, 9.4–18.8)	
		Total	366	165	201	
	**Emergency departments (ED) and walk in centres**	**Yes**	**323 (88.3, 84.5–91.2)**	**147 (89.1, 83.4–93.0)**	**176 (87.6, 82.3–91.4)**	0.565
		No	17 (4.6, 2.9–7.3)	6 (3.6, 1.7–7.7)	11 (5.5, 3.1–9.5)	
		Don’t know	26 (7.1, 4.9–10.2)	12 (7.3, 4.2–12.3)	14 (7.0, 4.2–11.4)	
		Total	366	165	201	
**Which of the following would be a motivation or incentive to test for BBVs?**	**Performance payment structure**	**Yes**	**181 (76.1, 70.2–81.0)**	**91 (74.6, 66.2–81.5)**	**90 (77.6, 69.2–84.2)**	0.588
		No	21 (8.8, 5.8–13.1)	15 (12.3, 7.6–19.3)	6 (5.2, 2.4–10.8)	
		Don’t know	36 (15.1, 11.1–20.2)	16 (13.1, 8.2–20.2)	20 (17.2, 11.4–25.1)	
		Total	238	122	116	
	**Local targets**	**Yes**	**164 (69.2, 63.1–74.7)**	**74 (61.2, 52.3–69.4)**	**90 (77.6, 69.2–84.2)**	0.006
		No	41 (17.3, 13.0–22.6)	31 (25.6, 18.7–34.1)	10 (8.6, 4.7–15.1)	
		Don’t know	32 (13.5, 9.7–18.4)	16 (13.2, 8.3–20.4)	16 (13.8, 8.7–21.2)	
		Total	237	121	116	
	**National goals**	**Yes**	**133 (56.1, 49.8–62.3)**	**56 (46.3, 37.6–55.1)**	**77 (66.4, 57.4–74.3)**	0.002
		No	58 (24.5, 19.4–30.3)	45 (37.2, 29.1–46.1)	13 (11.2, 6.7–18.2)	
		Don’t know	46 (19.4, 14.9–24.9)	20 (16.5, 11.0–24.2)	26 (22.4, 15.8–30.8)	
		Total	237	121	116	
	**CCG recommendations**	**Yes**	**174 (73.1, 67.1–78.3)**	**80 (65.6, 56.8–73.4)**	**94 (81.0, 73.0–87.1)**	0.007
		No	35 (14.7, 10.8–19.8)	28 (23.0, 16.4–31.2)	7 (6.0, 3.0–11.9)	
		Don’t know	29 (12.2, 8.6–17.0)	14 (11.5, 7.0–18.3)	15 (12.9, 8.0–20.2)	
		Total	238	122	116	
	**PHE recommendations**	**Yes**	**174 (73.1, 67.1–78.3)**	**84 (68.9, 60.2–76.4)**	**90 (77.6, 69.2–84.2)**	0.129
		No	29 (12.2, 8.6–17.0)	21 (17.2, 11.5–24.9)	8 (6.9, 3.5–13.0)	
		Don’t know	35 (14.7, 10.8–19.8)	17 (13.9, 8.9–21.2)	18 (15.5, 10.0–23.2)	
		Total	238	122	116	
	**NICE guidance**	**Yes**	**161 (67.4, 61.2–73.0)**	**75 (61.0, 52.1–69.1)**	**86 (74.1, 65.5–81.2)**	0.03
		No	39 (16.3, 12.2–21.5)	31 (25.2, 18.4–33.5)	8 (6.9, 3.5–13.0)	
		Don’t know	39 (16.3, 12.2–21.5)	17 (13.8, 8.8–21.0)	22 (19.0, 12.9–27.0)	
		Total	239	123	116	
	**NHSE recommendations**	**Yes**	**105 (44.5, 38.3–50.9)**	**51 (42.5, 34.0–51.4)**	**54 (46.6, 37.7–55.6)**	0.531
		No	50 (21.2, 16.5–26.8)	39 (32.5, 24.8–41.3)	11 (9.5, 5.4–16.2)	
		Don’t know	81 (34.3, 28.6–40.6)	30 (25.0, 18.1–33.4)	51 (44.0, 35.3–53.0)	
		Total	236	120	116	
	**CMO letter**	**Yes**	**104 (43.7, 37.5–50.0)**	**50 (41.0, 32.7–49.9)**	**54 (46.6, 37.7–55.6)**	0.387
		No	53 (22.3, 17.4–28.0)	39 (32.0, 24.4–40.7)	14 (12.1, 7.3–19.2)	
		Don’t know	81 (34.0, 28.3–40.3)	33 (27.0, 20.0–35.5)	48 (41.4, 32.8–50.5)	
		Total	238	122	116	
**What tests are requested when you test for hepatitis B?**		HBsAg + HBcAb	76 (33.9, 28.0–40.4)	39 (35.8, 27.4–45.1)	37 (32.2, 24.3–41.2)	0.138
		HBsAg	73 (32.6, 26.8–39.0)	36 (33.0, 24.9–42.3)	38 (33.0, 25.1–42.1)	
		HBcAb	10 (4.5, 2.4–8.0)	5 (4.6, 2.0–10.3)	5 (4.3, 1.9–9.8)	
		**Not known**	**45 (20.1, 15.4–25.8)**	**17 (15.6, 10.0–23.6)**	**27 (23.5, 16.7–32.0)**	
		Other, please specify	20 (8.9, 5.9–13.4)	12 (11.0, 6.4–18.3)	8 (7.0, 3.6–13.1)	
		Total	224	109	115	
**What tests are requested when you test for HCV?**		Anti-HCV + HCV RNA	32 (14.3, 10.3–19.5)	17 (15.6, 10.0–23.6)	15 (13.0, 8.1–20.4)	0.007
		Anti-HCV	56 (25.0, 19.8–31.1)	36 (33.0, 24.9–42.3)	20 (17.4, 11.5–25.3)	
		HCV RNA	24 (10.7, 7.3–15.4)	11 (10.1, 5.7–17.2)	13 (11.3, 6.7–18.4)	
		**Not known**	**86 (38.4, 32.3–44.9)**	**32 (29.4, 21.6–38.5)**	**54 (47.0, 38.1–56.0)**	
		Other, please specify	26 (11.6, 8.0–16.5)	13 (11.9, 7.1–19.3)	13 (11.3, 6.7–18.4)	
		Total	224	109	115	
**What tests are requested when you test for HIV?**		Anti-HIV	118 (52.7, 46.2–59.1)	72 (66.1, 56.8–74.3)	47 (40.9, 32.3–50.0)	< 0.0001
		**Not known**	**71 (31.7, 26.0–38.1)**	**20 (18.3, 12.2–26.6)**	**50 (43.5, 34.8–52.6)**	
		Other, please specify	35 (15.6, 11.5–21.0)	17 (15.6, 10.0–23.6)	18 (15.7, 10.1–23.4)	
		Total	224	109	115	
**Do you use Dried Blood Spot (DBS) testing?**		**Yes**	**21 (9.4, 6.2–13.9)**	**6 (5.5, 2.5–11.5)**	**15 (13.0, 8.1–20.4)**	
		No	172 (76.8, 70.8–81.8)	89 (81.7, 73.4–87.8)	83 (72.2, 63.4–79.5)	
		Don’t know	31 (13.8, 9.9–19.0)	15 (13.8, 8.5–21.5)	16 (13.9, 8.7–21.4)	
		Total	224	109	115	
**These questions relate to diagnosed patients**	**Are all patients with hepatitis B referred to secondary care?**	**Yes**	**176 (78.9, 73.1–83.8)**	**99 (91.7, 84.9–95.6)**	**77 (67.0, 57.9–74.9)**	< 0.0001
		No	13 (5.8, 3.4–9.7)	8 (7.4, 3.8–13.9)	5 (4.3, 1.9–9.8)	
		Don’t know	34 (15.2, 11.1–20.5)	1 (0.9, 0.2–5.1)	33 (28.7, 21.2–37.5)	
		Total	223	108	115	
	**Are all patients with hepatitis C referred to secondary care?**	**Yes**	**180 (80.7, 75.0–85.4)**	**102 (94.4, 88.4–97.4)**	**78 (67.8, 58.8–75.7)**	< 0.0001
		No	9 (4.0, 2.1–7.5)	5 (4.6, 2.0–10.4)	4 (3.5, 1.4–8.6)	
		Don’t know	34 (15.2, 11.1–20.5)	1 (0.9, 0.2–5.1)	33 (28.7, 21.2–37.5)	
		Total	223	108	115	
	**Do you receive information or check whether a patient has attended?**	**Yes**	**123 (55.2, 48.6–61.5)**	**67 (62.0, 52.6–70.6)**	**56 (48.7, 39.8–57.7)**	0.045
		No	37 (16.6, 12.3–22.0)	26 (24.1, 17.0–32.9)	11 (9.6, 5.4–16.3)	
		Don’t know	63 (28.3, 22.7–34.5)	15 (13.9, 8.6–21.7)	48 (41.7, 33.1–50.9)	
		Total	223	108	115	
	**Do you follow up patients that have not attended secondary care appointments?**	**Yes**	**104 (46.6, 40.2–53.2)**	**57 (52.8, 43.4–61.9)**	**47 (40.9, 32.3–50.0)**	0.075
		No	51 (22.9, 17.8–28.8)	37 (34.3, 26.0–43.6)	14 (12.2, 7.4–19.4)	
		Don’t know	68 (30.5, 24.8–36.8)	14 (13.0, 7.9–20.6)	54 (47.0, 38.1–56.0)	
		Total	223	108	115	
	**Are close contacts of HBV cases offered testing?**	**Yes**	**161 (72.2, 66.0–77.7)**	**80 (74.1, 65.1–81.4)**	**81 (70.4, 61.5–78.0)**	0.544
		No	13 (5.8, 3.4–9.7)	9 (8.3, 4.4–15.1)	4 (3.5, 1.4–8.6)	
		Don’t know	49 (22.0, 17.0–27.9)	19 (17.6, 11.6–25.8)	30 (26.1, 18.9–34.8)	
		Total	223	108	115	
	**Are close contacts of HBV cases offered vaccination?**	**Yes**	**165 (74.0, 67.9–79.3)**	**79 (73.1, 64.1–80.6)**	**86 (74.8, 66.1–81.8)**	0.781
		No	14 (6.3, 3.8–10.3)	9 (8.3, 4.4–15.1)	5 (4.3, 1.9–9.8)	
		Don’t know	44 (19.7, 15.0–25.4)	20 (18.5, 12.3–26.9)	24 (20.9, 14.4–29.2)	
		Total	223	108	115	

Abbreviation Definition

anti-HCV Hepatitis C antibody

anti-HIV HIV antibodies

BBV Blood borne virus

CCG Clinical commissioning group

CMO Chief Medical Officer

HBcAb Hepatitis B core antibody

HBsAg Hepatitis B surface antigen

HBV Hepatitis B virus

HCV Hepatitis C virus

HCV RNA Hepatitis C virus ribonucelic acid

HIV Human immunideficiency virus

NHSE NHS England

NICE National Institute for Health and Care Excellence

PHE Public Health England

Responses in bold indicate response used when creating dichotomised responses for Chi squared tests

### Knowledge of migrants’ entitlements to health services

When asked about migrants’ entitlements to health services (100% response rate), 88.3% (323/366, 84.5–91.2) of clinical staff correctly identified emergency departments and walk-in centres as free to all and 76.0% (279/367, 71.4–80.1) correctly identified GP and nurse consultations in primary care as free to all, with no difference by profession (*p* = 0.565 and 0.262 respectively, Table 2). Surgery or outpatient services in secondary care were correctly identified as not free to all by 52.3% (192/367, 47.2–57.4), and this was higher for GPs (59.4%, 98/165, 51.8–66.6) than nurses (46.5%, 94/202, 39.8–53.4) (*p* = 0.014).

### Laboratory tests for BBV diagnosis

When testing for HBV (64% response rate), 32.6% (73/224, 26.8–39.0) of clinical staff requested tests for hepatitis B surface antigen (HBsAg) only, 33.9% (76/224, 28.0–40.4) requested both HBsAg and hepatitis B core antibody (HBcAb) testing, 4.5% (10/224, 2.4–8.0) tested for HBcAb only, 20.1% (45/224, 15.4–25.8) did not know what test was requested and 8.9% (20/224, 5.9–13.4) stated ‘other,’ with most in this group stating that the laboratory determined the tests (Table 2). These results did not differ by profession (*p* = 0.138).

For HCV (64% response rate), 33.0% (36/109, 24.9–44.3) GPs and 17.4% (20/115, 11.5–25.3) nurses tested for hepatitis C antibody (anti-HCV) only, 15.6% (17/109, 10.0–23.6) GPs and 13.0% (15/115, 8.1–20.4) nurses tested for both anti-HCV and HCV RNA, while 10.1% (11/109, 5.7–17.2) GPs and 11.3% (13/115, 6.7–18.4) tested for HCV RNA only. There were 29.4% (32/109, 21.6–38.5) of GPs and 47.0% (54/115, 38.1–56.0) of nurses who did not know which tests were ordered (*p* = 0.007), and 11.6% (26/224, 8.0–16.5) stated ‘other’; most of these stated that the laboratory determined the test, or that a tick-box ‘hep C screen’ was completed.

For HIV (64% response rate), 66.1% (72/109, 56.8–74.3) GPs and 40.9% (47/115, 32.3–50.0) nurses tested for HIV antibodies, 18.3% (20/109, 12.2–26.6) GPs and 43.5% (50/115, 34.8–52.6) nurses did not know what tests were ordered, and 15.6% (35/224, 11.5–21.0) stated ‘other.’ Significantly more nurses than GPs did not know which tests were required for diagnosis of HCV and HIV (*p* < 0.01).

### Incentives for testing

Performance payment structure, PHE recommendations, Clinical Commissioning Group (CCG) recommendations, local targets, NICE recommendations and national goals were all endorsed as incentives or motivators to test migrants for BBVs by over 50% of clinical staff (65% response rate) (Table 2). Performance payment structure (76.1%, 181/238, 70.2–81.0) had the greatest reported influence on testing, followed by PHE recommendations and CCG recommendations (both 73.1%, 174/238, 67.1–78.3). Chief Medical Officer (CMO) recommendations (43.7%, 104/238, 37.5–50.0) and NHS England (NHSE) recommendations (44.5%, 105/235, 38.3–50.9) had the least. CCG recommendations, local targets, NICE guidance and national goals were all endorsed by a significantly higher proportion of nurses than GPs as incentives or motivators (*p* < 0.05).

### Management of patients diagnosed with hepatitis B and C

All persons positive for HBV were referred to secondary care by 91.7% (99/108, 84.9–95.6) of GPs and 67.0% (77/115, 57.9–74.9) of nurses (*p* < 0.01) and this was 94.4% (102/108, 88.4–97.4) and 67.8% (78/115, 58.8–75.7) respectively for persons positive for HCV (p < 0.01), with a higher proportion of nurses (28.7%, 33/115, 21.2–37.5) than GPs (0.9%, 1/108, 0.2–5.1) reporting they didn’t know for both questions) (p < 0.01, 54% response rate) (Table 2). Information on whether the patient had attended was received by 62.0% (67/108, 52.6–70.6) of GPs and 48.7% (56/115, 39.8–57.7) of nurses (p < 0.01) and 52.8% (57/108, 43.4–61.9) of GPs and 40.9% (47/115, 32.3–50.0) of nurses followed up patients who had not attended (p < 0.01). Close contacts of HBV infected cases were offered testing by 74.1% (80/108, 65.1–81.4) of GPs and 70.4% (81/115, 61.5–78.0) of nurses (*p* = 0.544), and vaccination by 73.1% (79/108, 64.1–80.6) and 74.8% (86/115, 66.1–81.8) respectively (*p* = 0.781).

## Practice policy

### Practice policy for offering BBV testing to migrant patients

HBV testing was offered on a universal/opt-out basis to all newly registering migrant patients by 17.8% (46/258, 13.6–23.0) of respondents, with 17.1% (44/257, 13.0–22.2) for HCV and 20.9% (54/258, 16.4–26.4) for HIV (all 62% response rate, *p* = 0.498) (Table 3). For existing patients who were migrants this was significantly lower: 11.2% (29/258, 7.9–15.7) for HBV (*p* = 0.045), 10.7% (27/252, 7.5–15.1) for HCV (*p* = 0.041) and 14.0% (36/258, 10.3–18.7) for HIV (*p* = 0.048) (62% response rate for HBV and HIV, 61% for HCV). Universal opt-out testing for newly registering migrant patients was significantly higher where respondents saw migrant patients frequently; 36.2% (34/94, 27.2–46.2) for HBV, 35.5% (33/93, 26.5–45.6) for HCV and 41.5% (39/54, 32.1–51.6) for HIV than where they saw them sometimes or rarely; 7.9% (13/164, 4.7–13.1) for HBV, 7.3% (12/164, 4.2–12.4) for HCV and 9.8% (16/164, 6.1–15.3) for HIV, *p* < 0.0001), and was higher in small practices, with a similar pattern for existing migrant patients.

**Table 3:** Primary care practice policy for blood borne virus (BBV) testing for migrant patients

Question	Blood borne virus (BBV)	Total Number (%, 95CI)	P (difference between questions)	By frequency of seeing migrant patients			By practice size			
				Frequently Number (%, 95CI)	Sometimes or rarely Number (%, 95CI)	P (frequency of seeing migrant patients)	Number (%, 95CI)	Number (%, 95CI)	Number (%, 95CI)	P (practice size)
**What is your practice policy for offering blood borne virus (BBV) testing to new migrants?**	**Hepatitis B**		HBV new vs existing migrants p = 0.045			< 0.0001				0.001
	**Universal / ‘opt out’**	**46 (17.8, 13.6–23.0)**		**34 (36.2, 27.2–46.2)**	**13 (7.9, 4.7–13.1)**		**16 (42.1, 27.9–57.8)**	**6 (14.3, 6.7–27.8)**	**9 (12.9, 6.9–22.7)**	
	Ad hoc	113 (43.8, 37.9–49.9)		37 (39.4, 30.1–49.5)	76 (46.3, 38.9–54.0)		11 (28.9, 17.0–44.8)	18 (42.9, 29.1–57.8)	31 (44.3, 33.2–55.9)	
	Other	18 (7.0, 4.5–10.8)		5 (5.3, 2.3–11.9)	12 (7.3, 4.2–12.4)		4 (10.5, 4.2–24.1)	2 (4.8, 1.3–15.8)	6 (8.6, 4.0–17.5)	
	Don’t know	81 (31.4, 26.0–37.3)		18 (19.1, 12.5–28.3)	63 (38.4, 31.3–46.0)		7 (18.4, 9.2–33.4)	16 (38.1, 25.0–53.2)	24 (34.3, 24.2–46.0)	
	Total responses	258		94	164		38	42	70	
	**Hepatitis C**		HCV new vs existing migrants p = 0.041			< 0.0001				0.001
	**Universal / ‘opt out’**	**44 (17.1, 13.0–22.2)**		**33 (35.5, 26.5–45.6)**	**12 (7.3, 4.2–12.4)**		**15 (39.5, 25.6–55.3)**	**6 (14.3, 6.7–27.8)**	**8 (11.6, 6.0–21.2)**	
	Ad hoc	114 (44.4, 38.4–50.5)		37 (39.8, 30.4–49.9)	77 (47.0, 39.5–54.6)		12 (31.6, 19.1–47.5)	18 (42.9, 29.1–57.8)	31 (44.9, 33.8–56.6)	
	Other	18 (7.0, 4.5–10.8)		5 (5.4, 2.3–12.0)	12 (7.3, 4.2–12.4)		4 (10.5, 4.2–24.1)	2 (4.8, 1.3–15.8)	6 (8.7, 4.0–17.7)	
	Don’t know	81 (31.5, 26.1–37.4)		18 (19.4, 12.6–28.5)	63 (38.4, 31.3–46.0)		7 (18.4, 9.2–33.4)	16 (38.1, 25.0–53.2)	24 (34.8, 24.6–46.6)	
	Total responses	257		93	164		38	42	69	
	**HIV**		HIV vs HBV, HCV for new migrants p = 0.498 HIV new vs existing migrants p = 0.048			< 0.0001				0.002
	**Universal / ‘opt out’**	**54 (20.9, 16.4–26.3)**		**39 (41.5, 32.1–51.6)**	**16 (9.8, 6.1–15.3)**		**18 (47.4, 32.5–62.7)**	**7 (16.7, 8.3–30.6)**	**14 (20.0, 12.3–30.8)**	
	Ad hoc	111 (43.0, 37.1–49.1)		35 (37.2, 28.1–47.3)	76 (46.3, 38.9–54.0)		10 (26.3, 15.0–42.0)	18 (42.9, 29.1–57.8)	29 (41.4, 30.6–53.1)	
	Other	17 (6.6, 4.2–10.3)		5 (5.3, 2.3–11.9)	11 (6.7, 3.8–11.6)		4 (10.5, 4.2–24.1)	2 (4.8, 1.3–15.8)	5 (7.1, 3.1–15.7)	
	Don’t know	76 (29.5, 24.2–35.3)		15 (16.0, 9.9–24.7)	61 (37.2, 30.2–44.8)		6 (15.8, 7.4–30.4)	15 (35.7, 23.0–50.8)	22 (31.4, 21.8–43.0)	
	Total responses	258		94	164		38	42	70	
**What is your practice policy for offering BBV testing to existing migrants?**	**Hepatitis B**		HIV vs HBV, HCV for existing migrants *p* = 0.480			< 0.0001				0.001
	**Universal / ‘opt out’**	**29 (11.2, 7.9–15.7)**		**21 (22.3, 15.1–31.8)**	**8 (4.9, 2.5–9.3)**		**11 (28.9, 17.0–44.8)**	**3 (7.1, 2.5–19.0)**	**3 (4.3, 1.5–11.9)**	
	Ad hoc	133 (51.6, 45.5–57.6)		46 (48.9, 39.1–58.9)	87 (53.0, 45.4–60.5)		17 (44.7, 30.1–60.3)	21 (50.0, 35.5–64.5)	39 (55.7, 44.1–66.8)	
	Other	25 (9.7, 6.6–13.9)		11 (11.7, 6.7–19.8)	14 (8.5, 5.2–13.8)		5 (13.2, 5.8–27.3)	4 (9.5, 3.8–22.1)	7 (10.0, 4.9–19.2)	
	Don’t know	71 (27.5, 22.4–33.3)		16 (17.0, 10.8–25.9)	55 (33.5, 26.8–41.1)		5 (13.2, 5.8–27.3)	14 (33.3, 21.0–48.4)	21 (30.0, 20.5–41.5)	
	Total	258		94	164		38	42	70	
	**Hepatitis C**					< 0.0001				< 0.0001
	**Universal / ‘opt out’**	**27 (10.7, 7.5–15.1)**		**20 (21.5, 14.4–30.9)**	**7 (4.4, 2.1–8.8)**		**11 (30.6, 18.0–46.9)**	**2 (5.0, 1.4–16.5)**	**2 (2.9, 0.8–10.0)**	
	Ad hoc	131 (52.0, 45.8–58.1)		46 (49.5, 39.5–59.4)	85 (53.5, 45.7–61.0)		16 (44.4, 29.5–60.4)	20 (50.0, 35.2–64.8)	39 (56.5, 44.8–67.6)	
	Other	25 (9.9, 6.8–14.2)		11 (11.8, 6.7–19.9)	14 (8.8, 5.3–14.2)		5 (13.9, 6.1–28.7)	4 (10.0, 4.0–23.1)	7 (10.1, 5.0–19.5)	
	Don’t know	69 (27.4, 22.2–33.2)		16 (17.2, 10.9–26.1)	53 (33.3, 26.5–41.0)		4 (11.1, 4.4–25.3)	14 (35.0, 22.1–50.5)	21 (30.4, 20.8–42.1)	
	Total	252		93	159		36	40	69	
	**HIV**					< 0.0001				< 0.0001
	**Universal / ‘opt out’**	**36 (14.0, 10.3–18.7)**		**24 (25.5, 17.8–35.2)**	**12 (7.3, 4.2–12.4)**		**14 (36.8, 23.4–52.7)**	**4 (13.8, 5.5–30.6)**	**5 (9.8, 4.3–21.0)**	
	Ad hoc	133 (51.6, 45.5–57.6)		46 (48.9, 39.1–58.9)	87 (53.0, 45.4–60.5)		16 (42.1, 27.9–57.8)	21 (72.4, 54.3–85.3)	39 (76.5, 63.2–86.0)	
	Other	25 (9.7, 6.6–13.9)		11 (11.7, 6.7–19.8)	14 (8.5, 5.2–13.8)		5 (13.2, 5.8–27.3)	4 (13.8, 5.5–30.6)	7 (13.7, 6.8–25.7)	
	Don’t know	64 (24.8, 19.9–30.4)		13 (13.8, 8.3–22.2)	51 (31.1, 24.5–38.5)		3 (7.9, 2.7–20.8)	13 (44.8, 28.4–62.5)	19 (37.3, 25.3–51.0)	
	Total	258		94	164		38	29	51	
**How are existing migrants identified for testing?**	**Opportunistic during consultation**		n/a			0.052				0.111
	**Yes**	**191 (78.9, 73.4–83.6)**		**77 (85.6, 76.8–91.4)**	**114 (75.0, 67.6–81.2)**		**34 (89.5, 75.9–95.8)**	**30 (71.4, 56.4–82.8)**	**53 (75.7, 64.5–84.2)**	
	No	9 (3.7, 2.0–6.9)		5 (5.6, 2.4–12.4)	4 (2.6, 1.0–6.6)		1 (2.6, 0.5–13.5)	2 (4.8, 1.3–15.8)	4 (5.7, 2.2–13.8)	
	Don’t know	42 (17.4, 13.1–22.6)		8 (8.9, 4.6–16.6)	34 (22.4, 16.5–29.6)		3 (7.9, 2.7–20.8)	10 (23.8, 13.5–38.5)	13 (18.6, 11.2–29.2)	
	Total	242		90	152		38	7	70	
	**Any periodic flagging**		n/a			0.026				0.04
	**Yes**	**33 (13.6, 9.9–18.5)**		**18 (20.0, 13.0–29.4)**	**15 (9.9, 6.1–15.6)**		**10 (26.3, 15.0–42.0)**	**4 (9.5, 3.8–22.1)**	**7 (10.0, 4.9–19.2)**	
	No/don’t know	209 (86.4, 81.5–90.1)		72 (80.0, 70.6–87.0)	137 (90.1, 84.4–93.9)		28 (73.7, 58.0–85.0)	38 (90.5, 77.9–96.2)	63 (90.0, 80.8–95.1)	
	Total	242		90	152		38	42	70	

Responses in bold indicate response used when creating dichotomised responses for Chi squared tests

Testing practices varied by PHE region. Practice policy for offering universal opt-out HIV testing for newly registering migrant patients ranged from 53.1% (17/32, 36.4–69.1) of respondents in Yorkshire and the Humber to 4.9% (2/41, 1.3–6.1) of respondents in the South East (p < 0.0001), and this regional pattern was similar for HBV and HCV (supplementary Table 1). For existing patients who were migrants, universal opt-out testing was highest in the Midlands (31.7%, 13/41, 19.6–47.0 for HIV) and lowest in the South East (4.9%, 2/41, 1.3–16.1 for HIV), *p* < 0.05 for all three BBV.

### Identification of migrant patients for BBV testing

When asked if existing migrant patients were identified for testing opportunistically during consultation, 78.9% (191/242, 73.4–83.6) of respondents stated they were (61% response rate), while only 13.6% (33/242, 9.9–18.5) stated that systematic identification methods, either periodic flagging of the GP system by automated software, or periodic manual audit of the GP system, were used (61% response rate) (Table 3). Flagging was significantly higher among those who saw migrant patients frequently (20.0%, 18/90, 13.0–29.4) than those who saw migrant patients sometimes/rarely (9.9%, 15/152, 6.1–15.6, *p* = 0.026), and in small practices (26.3%, 10/38, 15.0–42.0) compared to medium (9.5%, 4/42, 3.8–22.1) or large (10.0%, 7/70, 4.9–19.2) (*p* = 0.04) and was highest in Yorkshire and the Humber (25.0%, 8/32, 13.3–42.1) and London (22.5%, 9/40, 12.3–37.5) and lowest in the South East (0.0%, 0/15, 0.0–20.4), South West (2.6%, 1/38, 0.5–13.5), and North West (3.1%, 1/32, 0.6–15.7) (*p* = 0.012) (supplementary Table 1).

### Services offered

Most (87.5%, 133/152, 81.3–91.8) practices provided interpreter services (38% response rate), and 22.0% (53/241, 17.2–27.6) offered longer appointments to migrants at registration (61% response rate), but few offered other services to facilitate BBV testing and care of migrants (Table 4): 9.5% (23/241, 6.4–13.9) had a specially designated clinic; 8.7% (21/241, 5.8–13.0) had a designated GP; 11.6% (28/241, 8.2–16.3) had specific projects to register migrants; 8.3% (20/241, 5.4–12.5) had outreach facilities, 7.5% (18/241, 4.8–11.5) had health support teams and 4.6% (11/241, 2.6–8.0) had incentive schemes for GPs. Outreach services (*p* = 0.024) and projects to register migrants (*p* = 0.019) were both more common in small practices, and projects to register new migrants, specially designated clinics, longer appointments at registration (all *p* < 0.01) and outreach services (*p* = 0.029) were all more common in practices where respondents saw migrant patients frequently. Provision of longer appointments at registration varied significantly by region and was highest in Yorkshire and Humber (46.9%, 15/32, 30.9–63.6), followed by the North East (41.7%, 5/12, 19.3–68.0) and was lowest in the North West (6.3%, 2/32, 1.7–20.1) (p < 0.01) (supplementary Table 2).

**Table 4:** Primary care practice facilities and practice policy for recording data

Question		No. (%, 95%CI)	P (between questions)	By frequency of seeing migrant patients			By practice size			
				Frequently No. (%, 95%CI)	Sometimes or rarely No. (%, 95%CI)	P (frequency of seeing migrant patients)	Small No. (%, 95%CI)	Med No. (%, 95%CI)	Large No. (%, 95%CI)	P (practice size)
**Services in place to facilitate blood borne virus (BBV) testing and care of migrant patients**	**Interpreter facilities**		n/a			0.266				0.568
	**Yes**	**133 (87.5, 81.3-91.8)**		**60 (90.9, 81.6-95.8)**	**73 (84.9, 75.8-90.9)**		**35 (92.1, 79.2-97.3)**	**36 (87.8, 74.5-94.7)**	**59 (84.3, 74.0-91.0)**	
	No	14 (9.2, 5.6-14.9)		5 (7.6, 3.3-16.5)	9 (10.5, 5.6-18.7)		2 (5.3, 1.5-17.3)	4 (9.8, 3.9-22.5)	8 (11.4, 5.9-21.0)	
	Don’t know	5 (3.3, 1.4-7.5)		1 (1.5, 0.3-8.1)	4 (4.7, 1.8-11.4)		1 (2.6, 0.5-13.5)	1 (2.4, 0.4-12.6)	3 (4.3, 1.5-11.9)	
	Total responses	152		66	86		38	41	70	
	**Longer appointments at registration**		n/a			< 0.0001				0.083
	**Yes**	**53 (22.0, 17.2-27.6)**		**31 (34.4, 25.4-44.7)**	**22 (14.6, 9.8-21.1)**		**14 (36.8, 23.4-52.7)**	**10 (23.8, 13.5-38.5)**	**12 (17.1, 10.1-27.6)**	
	No	162 (67.2, 61.1-72.8)		53 (58.9, 48.6-68.5)	109 (72.2, 64.6-78.7)		22 (57.9, 42.2-72.1)	29 (69.0, 54.0-80.9)	52 (74.3, 63.0-83.1)	
	Don’t know	26 (10.8, 7.5-15.3)		6 (6.7, 3.1-13.8)	20 (13.2, 8.7-19.6)		2 (5.3, 1.5-17.3)	3 (7.1, 2.5-19.0)	6 (8.6, 4.0-17.5)	
	Total responses	241		90	151		38	42	70	
	**Specially designated clinic**		n/a			< 0.0001				0.074
	**Yes**	**23 (9.5, 6.4-13.9)**		**17 (18.9, 12.1-28.2)**	**6 (4.0, 1.8-8.4)**		**6 (15.8, 7.4-30.4)**	**1 (2.4, 0.4-12.3)**	**4 (5.7, 2.2-13.8)**	
	No	196 (81.3, 75.9-85.7)		69 (76.7, 66.9-84.2)	127 (84.1, 77.4-89.1)		30 (78.9, 63.7-88.9)	39 (92.9, 81.0-97.5)	61 (87.1, 77.3-93.1)	
	Don’t know	22 (9.1, 6.1-13.4)		4 (4.4, 1.7-10.9)	18 (11.9, 7.7-18.1)		2 (5.3, 1.5-17.3)	2 (4.8, 1.3-15.8)	5 (7.1, 3.1-15.7)	
	Total responses	241		90	151		38	42	70	
	**Designated GP**		n/a			0.05				1.00
	**Yes**	**21 (8.7, 5.8-13.0)**		**12 (13.3, 7.8-21.9)**	**9 (6.0, 3.2-10.9)**		**3 (7.9, 2.7-20.8)**	**3 (7.1, 2.5-19.0)**	**5 (7.1, 3.1-15.7)**	
	No	195 (80.9, 75.5-85.4)		71 (78.9, 69.4-86.0)	124 (82.1, 75.2-87.4)		32 (84.2, 69.6-92.6)	37 (88.1, 75.0-94.8)	61 (87.1, 77.3-93.1)	
	Don’t know	25 (10.4, 7.1-14.9)		7 (7.8, 3.8-15.2)	18 (11.9, 7.7-18.1)		3 (7.9, 2.7-20.8)	2 (4.8, 1.3-15.8)	4 (5.7, 2.2-13.8)	
	Total responses	241		90	151		38	42	70	
	**Specific projects to register migrants**		n/a			< 0.0001				0.024
	**Yes**	**28 (11.6, 8.2-16.3)**		**19 (21.1, 14.0-30.6)**	**9 (6.0, 3.2-10.9)**		**8 (21.1, 11.1-36.3)**	**1 (2.4, 0.4-12.3)**	**6 (8.6, 4.0-17.5)**	
	No	179 (74.3, 68.4-79.4)		63 (70.0, 59.9-78.5)	116 (76.8, 69.5-82.8)		28 (73.7, 58.0-85.0)	37 (88.1, 75.0-94.8)	57 (81.4, 70.8-88.8)	
	Don’t know	34 (14.1, 10.3-19.1)		8 (8.9, 4.6-16.6)	26 (17.2, 12.0-24.0)		2 (5.3, 1.5-17.3)	4 (9.5, 3.8-22.1)	7 (10.0, 4.9-19.2)	
	Total responses	241		90	151		38	42	70	
	**Outreach facilities**		n/a			0.029				0.019
	**Yes**	**20 (8.3, 5.4-12.5)**		**12 (13.3, 7.8-21.9)**	**8 (5.3, 2.7-10.1)**		**7 (18.4, 9.2-33.4)**	**3 (7.1, 2.5-19.0)**	**2 (2.9, 0.8-9.8)**	
	No	190 (78.8, 73.2-83.5)		73 (81.1, 71.8-87.9)	117 (77.5, 70.2-83.4)		27 (71.1, 55.2-83.0)	36 (85.7, 72.2-93.3)	63 (90.0, 80.8-95.1)	
	Don’t know	31 (12.9, 9.2-17.7)		5 (5.6, 2.4-12.4)	26 (17.2, 12.0-24.0)		4 (10.5, 4.2-24.1)	3 (7.1, 2.5-19.0)	5 (7.1, 3.1-15.7)	
	Total responses	241		90	151		38	42	70	
	**Outreach services specifically for migrants**		n/a			0.888				0.048
	**Yes**	**11 (7.2, 4.1-12.5)**		**5 (7.6, 3.3-16.5)**	**6 (7.0, 3.2-14.4)**		**6 (15.8, 7.4-30.4)**	**3 (7.3, 2.5-19.4)**	**2 (2.9, 0.8-9.8)**	
	No	111 (73.0, 65.5-79.4)		50 (75.8, 64.2-84.5)	61 (70.9, 60.6-79.5)		25 (65.8, 49.9-78.8)	31 (75.6, 60.7-86.2)	53 (75.7, 64.5-84.2)	
	Don’t know	30 (19.7, 14.2-26.8)		11 (16.7, 9.6-27.4)	19 (22.1, 14.6-31.9)		7 (18.4, 9.2-33.4)	7 (17.1, 8.5-31.3)	15 (21.4, 13.4-32.4)	
	Total responses	152		66	86		38	41	70	
	**Health support teams**		n/a			0.517				0.123
	**Yes**	**18 (7.5, 4.8-11.5)**		**8 (8.9, 4.6-16.6)**	**10 (6.6, 3.6-11.8)**		**5 (13.2, 5.8-27.3)**	**4 (9.5, 3.8-22.1)**	**2 (2.9, 0.8-9.8)**	
	No	184 (76.3, 70.6-81.3)		73 (81.1, 71.8-87.9)	111 (73.5, 66.0-79.9)		29 (76.3, 60.8-87.0)	35 (83.3, 69.4-91.7)	61 (87.1, 77.3-93.1)	
	Don’t know	39 (16.2, 12.1-21.4)		9 (10.0, 5.4-17.9)	30 (19.9, 14.3-26.9)		4 (10.5, 4.2-24.1)	3 (7.1, 2.5-19.0)	7 (10.0, 4.9-19.2)	
	Total responses	241		90	151		38	42	70	
	**Incentive scheme for GPs**		n/a			0.065				0.693
	**Yes**	**11 (4.6, 2.6-8.0)**		**7 (7.8, 3.8-15.2)**	**4 (2.6, 1.0-6.6)**		**1 (2.6, 0.5-13.5)**	**3 (7.1, 2.5-19.0)**	**3 (4.3, 1.5-11.9)**	
	No	184 (76.3, 70.6-81.3)		71 (78.9, 69.4-86.0)	113 (74.8, 67.4-81.1)		32 (84.2, 69.6-92.6)	32 (76.2, 61.5-86.5)	56 (80.0, 69.2-87.7)	
	Don't know	46 (19.1, 14.6-24.5)		12 (13.3, 7.8-21.9)	34 (22.5, 16.6-29.8)		5 (13.2, 5.8-27.3)	7 (16.7, 8.3-30.6)	11 (15.7, 9.0-26.0)	
	Total responses	241		90	151		38	42	70	
**Routine recording of data**	**Ethnicity**		Ethnicity vs country of birth *P* < 0.0001			0.168				0.939
	**Yes**	**137 (90.1, 84.4-93.9)**		**62 (93.9, 85.4-97.6)**	**75 (87.2, 78.5-92.7)**		**35 (92.1, 79.2-97.3)**	**37 (90.2, 77.5-96.1)**	**62 (88.6, 79.0-94.1)**	
	No	6 (3.9, 1.8-8.3)		1 (1.5, 0.3-8.1)	5 (5.8, 2.5-12.9)		3 (7.9, 2.7-20.8)	2 (4.9, 1.3-16.1)	1 (1.4, 0.3-7.7)	
	Don't know	9 (5.9, 3.1-10.9)		3 (4.5, 1.6-12.5)	6 (7.0, 3.2-14.4)		0 (0.0, 0.0-9.2)	2 (4.9, 1.3-16.1)	7 (10.0, 4.9-19.2)	
	Total responses	152		66	86		38	41	70	
	**Country of birth**					0.002				0.042
	**Yes**	**89 (58.6, 50.6-66.1)**		**48 (72.7, 61.0-82.0)**	**41 (47.7, 37.4-58.1)**		**28 (82.4, 66.5-91.7)**	**24 (66.7, 50.3-79.8)**	**34 (66.7, 53.0-78.0)**	
	No	35 (23.0, 17.0-30.3)		10 (15.2, 8.4-25.7)	25 (29.1, 20.5-39.4)		6 (17.6, 8.3-33.5)	12 (33.3, 20.2-49.7)	17 (33.3, 22.0-47.0)	
	Don't know	28 (18.4, 13.1-25.3)		8 (12.1, 6.3-22.1)	20 (23.3, 15.6-33.2)		4 (11.8, 4.7-26.6)	5 (13.9, 6.1-28.7)	19 (37.3, 25.3-51.0)	
	Total	152		66	86		34	36	51	
	**Hepatitis diagnosis**					0.064				0.711
	**Yes**	**113 (74.3, 66.9-80.6)**	n/a	**54 (81.8, 70.9-89.3)**	**59 (68.6, 58.2-77.4)**		**30 (78.9, 63.7-88.9)**	**29 (70.7, 55.5-82.4)**	**53 (75.7, 64.5-84.2)**	
	No	23 (15.1, 10.3-21.7)		7 (10.6, 5.2-20.3)	16 (18.6, 11.8-28.1)		6 (15.8, 7.4-30.4)	6 (14.6, 6.9-28.4)	10 (14.3, 7.9-24.3)	
	Don't know	16 (10.5, 6.6-16.4)		5 (7.6, 3.3-16.5)	11 (12.8, 7.3-21.5)		2 (5.3, 1.5-17.3)	6 (14.6, 6.9-28.4)	7 (10.0, 4.9-19.2)	
	Total responses	152		66	86		38	41	70	
	**Hepatitis prescription**					0.842				0.309
	**Yes**	**82 (53.9, 46.0-61.7)**	n/a	**35 (53.0, 41.2-64.6)**	**47 (54.7, 44.2-64.7)**		**24 (63.2, 47.3-76.6)**	**24 (58.5, 43.4-72.2)**	**34 (48.6, 37.2-60.0)**	
	No	40 (26.3, 20.0-33.8)		20 (30.3, 20.6-42.2)	20 (23.3, 15.6-33.2)		11 (28.9, 17.0-44.8)	9 (22.0, 12.0-36.7)	18 (25.7, 16.9-37.0)	
	Don't know	30 (19.7, 14.2-26.8)		11 (16.7, 9.6-27.4)	19 (22.1, 14.6-31.9)		3 (7.9, 2.7-20.8)	8 (19.5, 10.2-34.0)	18 (25.7, 16.9-37.0)	
	Total responses	152		66	86		38	41	70	

Responses in bold indicate response used when creating dichotomised responses for Chi squared tests

### Routine recording of data

Ethnicity was routinely recorded by 90.1% (137/152, 84.4–93.1) of respondents’ practices, whereas 58.6% (89/152, 50.6–66.1) reported that they routinely recorded country of birth (38% response rate), and this was significantly higher where respondents saw migrant patients frequently (p < 0.01) and in small practices (*p* = 0.042) (Table 4). Hepatitis diagnosis was routinely recorded by 74.3% (113/152, 66.9–80.3), and hepatitis prescription by 53.9% (82/152, 46.0–61.7) of respondents’ practices (Table 4).

### Perceived barriers for migrants to access healthcare across the care pathway

**Fig. 1:**
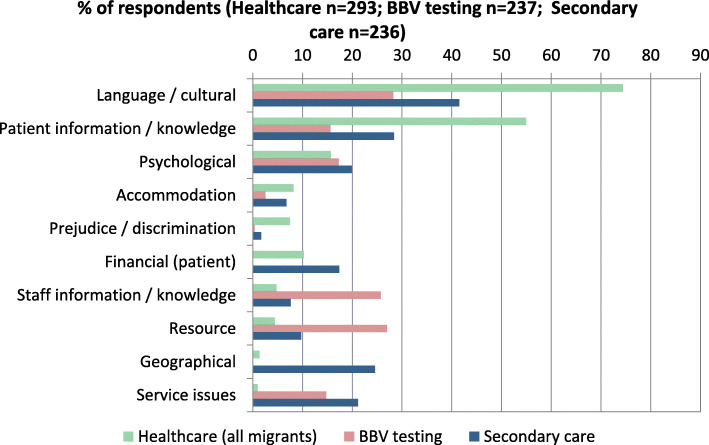
Perceived barriers for migrant populations to access different parts of the BBV care pathway, England, 2017

The content and proportion of responses in each theme varied according to which part of the care pathway was considered (Fig. 1, additional file 4).

For perceived barriers for migrants accessing healthcare (74% response rate), language and culture (predominantly language) were the most commonly reported (74.4%, 218/293, 69.1–79.1), followed by patient information and knowledge, particularly of the availability of health services and how to navigate the health system (54.9%, 161/293, 49.2–60.5), and psychological barriers, including fear (15.7%, 46/293, 12.0–20.3) (Fig. 1). Other common themes included accommodation or having no fixed abode (8.2%, 24/294, 5.5–11.9), prejudice and discrimination (7.5%, 22/293, 5.0–11.1), and patient financial barriers (10.2%, 30/293, 7.3–14.2).

Language and culture was also the most commonly cited barrier to accessing BBV services in primary care (28.3%, 67/237, 22.9–34.3, 60% response rate) followed by resource issues (27.0%, 64/237, 21.8–33.0), and staff knowledge and awareness (25.7%, 61/237, 20.6–31.7). Resource issues often related to time, whereas staff knowledge often related to a lack of staff training, or staff not being aware of patients’ migrant status. Psychological barriers including stigma and fear of diagnosis were cited by 17.3% (41/237, 13.0–226) of respondents, and service issues, such as practices not having a policy for BBV testing, or not identifying migrant patients, were given by 14.8% (35/237, 10.8–19.8) of respondents.

For patients to access BBV services in secondary care, again language and culture was the most common theme (41.5%, 98/236, 35.4–47.9, 60% response rate), followed by patient information and knowledge, with responses often relating to patients not understanding the significance of the diseases and importance of accessing treatment (28.4%, 67/236, 23.0–34.5). Geographical issues were cited by 24.5% (58/236, 19.5–30.4), including transport costs and lack of locally available services, and 21.2% (50/236, 16.5–26.8) cited service issues, including communication methods around arranging appointments, waiting times and lack of an organised clinic.

**Fig. 2:**
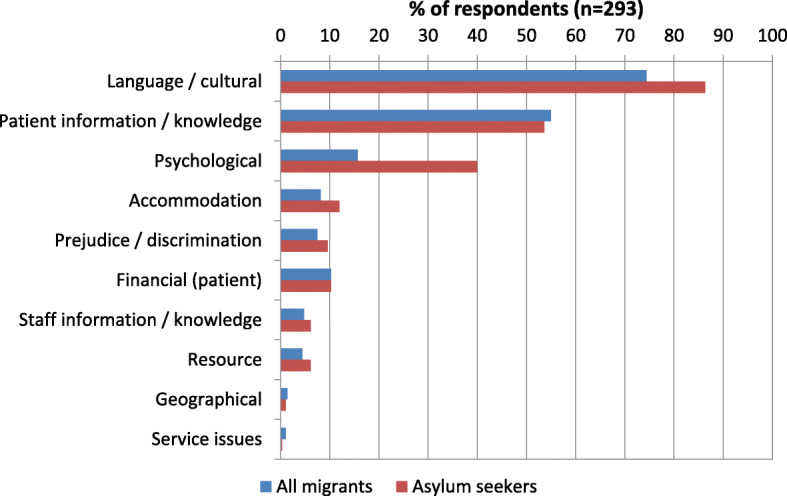
Perceived barriers to accessing health care for all migrants and for asylum seekers, England, 2017

Perceived barriers for asylum seekers accessing healthcare (74% response rate) were largely similar to those for all migrants, with some notable differences (Fig. 2). Language and psychological barriers were more frequently reported for asylum seekers than they were for all migrants. Psychological barriers again included fear, as well as highlighting mental health concerns for asylum seekers. To a lesser degree, accommodation, and prejudice and discrimination were also identified more often for asylum seekers than for all migrants.

## Discussion

Primary care staff knowledge, attitudes, policy and practice regarding migrants’ healthcare entitlements and their BBV testing and care was very variable in this sample of English practices. One in four were unaware that GP and nurse consultations are free for all. Universal opt-out BBV testing for migrants was not common practice, and only one in three clinical staff would routinely consider BBV risk assessments for newly registering migrant patients. Where testing was requested, most respondents requested the recommended tests, and over 90% indicated they would refer diagnosed cases to secondary care. Respondents identified access barriers relating to a range of factors including language; patient knowledge, stigma and fear; time pressures of appointments, practices not having a BBV testing policy and lack of knowledge and awareness among staff.

Knowledge of entitlements to health services varied. Of particular concern is that a quarter of clinical staff were unaware that GP and nurse consultations are free to all. Vulnerable patients, including migrants could be wrongly refused GP registration, despite national guidance that lack of documentation should not be a barrier to registering with a GP [24]. Three UK studies estimate that refusal ranges from 20 to 39% [25–27]. As primary care is the first point of access, ‘gatekeeper’ to other services, and a trusted source of health information, GP staff’s poor knowledge of entitlements is likely to create a further access barrier in addition to the system, cultural and language barriers migrants face, especially when it comes to communicable disease control [28, 29]. There were no substantial differences in knowledge of healthcare entitlements of migrants between GPs and nurses; GPs were more likely to know that operations and outpatient appointments are not free, and nurses were more likely to know that care for FGM, torture etc. is free, which likely reflects their roles in general practice.

BBV testing was more often conducted on an ad-hoc than opt-out basis, and BBV risk assessments for newly registering migrant patients were routinely considered by only a third of respondents, and more often considered on a situation specific basis. Most respondents stated existing migrant patients were identified for testing opportunistically during consultation, and only 14% systematically flagged migrant patients for BBV testing. These findings suggest that migrant patients eligible for BBV testing are not often routinely identified as such, which is likely to hinder adherence to BHIVA, ECDC and NICE guidelines which recommend BBV testing for migrant patients from higher HBV and HCV prevalence (> = 2%) areas and HIV testing for all patients in high (> 2 per 1000) prevalence areas in the UK [10, 11, 30], and is consistent with other studies which report low adherence to testing guidelines for BBVs outside of the sexual and antenatal health setting [12, 31]. Opt-out rapid testing in general practice has been shown to increase diagnosis rates for HIV, and universal opt-out testing initiatives for migrants which offer opportunities to normalise testing are subject to ongoing evaluations and have been well received by staff and patients [32–34].

Improved practice staff knowledge is needed to help reduce barriers and variations in service provision. Resources are available, such as the PHE Migrant Health Guide which outlines NHS entitlements and gives guidance on assessing new patients from overseas [35], RCGP e-learning courses [36], but also PHE and charities co-branded promotional videos, posters and leaflets on BBV in multiple languages for patient waiting areas can be downloaded or ordered from gov.uk [37]. Further work is needed to understand current awareness and to increase uptake of these resources.

Use of software programmes to identify and automatically flag patients for testing based on risk factors or risk proxies recorded on practice registers has been shown to be effective for groups including migrants in primary care and other settings [38–40]. This requires migrant status to be recorded on GP systems for migrants to be flagged as at risk. Although 59% of respondents stated that their practice routinely recorded country of birth, data from the Clinical Practice Research Datalink suggests recording is a lot lower at 1.6% [41]. This discrepancy could in part be due to biases in our sample or respondents giving socially desirable answers, however it may be that practices are recording this data on other systems where the data is not extractable. This highlights a need to increase awareness of the importance of recording patient country of birth amongst practitioners and information service providers, and for practitioners to assess risks associated with country of birth as a part of the first registration consultation.

Flagging and opt-out testing for newly registering migrant patients was more frequently reported where respondents said they see migrant patients frequently, in smaller practices and highest in Yorkshire and Humber and London, but lowest in the South East. A similar pattern was observed for services such as specially designated clinics, longer appointments at registration and projects to register migrants – these were more frequently reported where respondents saw migrants frequently. Outreach services and projects to register migrants were higher in small practices. These variations may reflect GP surgeries adopting policies and practices that reflect the needs and diversity of their local populations, and the ability of smaller practices to be more responsive and adaptive in implementing migrant focused services.

Performance payment structure may be the most influential way to incentivise BBV testing [42–44], and PHE and CCG recommendations were also more often stated to provide motivation to test than alternative sources. Only half of respondents indicated that NHSE and CMO recommendations would provide motivation for testing, which may reflect a relative lack of confidence in these bodies versus the perceived scientific credibility and independence of PHE and the more direct relationship and understanding of local PHE teams with CCGs. Interestingly, nurses were more likely than GPs to be motivated by local targets, national goals, CCG recommendations and NICE guidance.

Over 90% of GPs reported referring diagnosed cases of HBV and HCV to secondary care, and over half followed patients up if they did not attend secondary care appointments. Three quarters would offer close contacts of HBV infected cases testing, and three quarters would offer close contacts hepatitis B vaccination. These proportions seem high, as many patients diagnosed with HBV and HCV are known not to be accessing secondary care – in a 2012 UK study only a third of HBV diagnosed patients were on treatment [45] and an analysis of England sentinel surveillance data showed that between 2004 and 2017 only 21% of viraemic HCV diagnosed patients had evidence of treatment [46], and baseline HBV testing of close contacts was only 34% in a 2018 UK study [47]. Our results may be due to sampling and responder bias, due to initially contacting networks who were interested in migrant health, and those who were interested being more likely to respond, as well as wishing to give socially acceptable or desirable responses.

Differences in knowledge of BBV diagnosis and management were noted between nurses and GPs, with nurses being less likely to know whether all diagnosed HBV/HCV patients are referred and whether referrals are followed up, and less likely to know the diagnostic tests to request for HCV and HIV but there was no significant difference for HBV. Generally, there is a need for improved understanding of diagnostic testing for BBV in practices. Regardless of service, respondents cited language as the main barrier to accessing healthcare, which is consistent with the literature [14, 15, 48]. For patients who require linguistic support, interpreting services are recommended in General Medical Council guidance and were provided by 88% of respondents’ practices [49–51]. Nevertheless, there is evidence that interpreting services are underused, despite incurring no direct cost for practices [52]. This may be partly due to time pressures within appointments and the indirect costs of longer appointment times, as well as the need for forward planning and a lack of professional confidence in working with interpreters [53]. Brief education may help improve professional confidence in when and how to use interpreters [54]. Improved provision of translated materials is also likely to be beneficial in improving patient access to health information; efforts to ensure materials about BBV are translated are ongoing [55].

Patient knowledge and understanding of disease, and the location of secondary care services were cited as barriers to accessing secondary care. Community based treatment pathways, as have been demonstrated to be effective for HCV [56, 57], could help to overcome geographic barriers, although one recent study found that (first and second generation) migrant patients were no more likely to complete treatment on a community pathway [40]. Assessing patient attitudes towards BBV, and increasing patient knowledge would also be valuable and could be considered by practices to improve their services.

### Limitations

This survey has a low response rate and a relatively small sample size, and may be affected by sampling and responder bias, as the survey was disseminated by contacts and networks with an interest in migrant and asylum seeker health and through a publication which professionals with an interest in vaccines subscribe to, so practice professionals with interests in migrant health, BBVs and vaccine preventable diseases may have been more likely to respond. This is likely to bias the responses towards more knowledge of healthcare entitlements and better practices for BBV screening and management of migrants. Response to recruitment at professional conferences was poor, possibly due to competing priorities for professionals with limited time in a conference setting.

No demographic data on respondents was collected, so it was not possible to assess how responses vary by individual respondent characteristics, which may have associations with personal bias, stigma and discrimination against individuals who have migrated from specific countries or settings.

Our findings may therefore not be representative of all general practice staff. It was not possible to obtain data on non-respondents which would help to identify biases in responses; further work to survey practitioners within a fixed sampling frame (for example all GP practices in one CCG) may help to address this issue. In addition, not all questions were completed by all participants and fewer respondents completed later questions in the survey. Again, those more motivated may be more likely to complete the whole survey, leading to potentially greater bias in questions that had fewer respondents.

The small sample size limited our power to detect statistically significant differences between subgroups, particularly in detecting regional variations. Increasing the responses to the survey would help to address this, and in hindsight, following up the circulation of the survey link in Vaccine Updates could have helped to increase sample size and potentially reduce response bias.

Another limitation was that questions about barriers for migrant patients were asked to general practice professionals rather than migrants themselves; responses will reflect only barriers that these professionals were aware of and may exclude those which migrants experience, but professionals are less aware of. However, our findings echoed those cited by migrants in a qualitative study on barriers to accessing healthcare for viral hepatitis [14], which suggests that our respondents were relatively well informed about these issues.

## Conclusions

Systematic or universal opt-out testing for migrant patients are uncommon and testing is more often done on an ad hoc basis, despite BASHH, PHE and NICE guidelines. Achieving the WHO goal of eliminating viral hepatitis as a public health threat by 2030 requires coordinated efforts to increase case-finding of patients with HBV and HCV; our results suggest that current testing practices for high-risk migrants in primary care are likely to be inadequate to meet the elimination goal among this population. Improved systematic risk-based flagging of migrant patients for BBV screening and checks or prompts for referral and attendance to specialist services is needed to reduce the burden of undiagnosed infection and improve uptake of treatment, noting this is curative for hepatitis C. Local and national commissioning specifications for primary care testing and management of BBV infected patients may be required to achieve this goal. General practice professionals’ knowledge of migrants’ entitlements to healthcare was variable and could affect migrants’ access to care. Perceived barriers to accessing healthcare consistently included language and lack of patient and staff information and awareness. These are not insurmountable but require sustained commitment, professional awareness as well as resource. The ultimate goal is to reduce morbidity, mortality and inequalities associated with BBV infections in migrant populations, mindful of the fact that this population is already vulnerable to disparities in healthcare access and health outcomes.

## Supplementary Information

**Additional file 1.** Full online questionnaire. Text version of the online questionnaire used for the survey.

**Additional file 2.** Shorter paper questionnaire. PDF of the paper questionnaire that was distributed at the RCGP and Best Practice in Primary Care conferences.

**Additional file 3.** Definitions sheet which accompanied the questionnaire.

**Additional file 4.** Example responses for barriers questions. Example responses for free text barriers questions, by theme and part of care pathway.

## Data Availability

The datasets used and analysed during the current study are available from the corresponding author on reasonable request.

## Abbreviations

Anti-HCV: Antibodies against hepatitis C virus
BASHH: British Association of Sexual Health and HIV
BBV: Blood borne virus
BHIVA: British HIV Association
CCG: Clinical commissioning group
CMO: Chief Medical Officer
DBS: Dried blood spot
ECDC: European Centre for Disease Prevention and Control
ED: Emergency department
GP: General practitioner
HBcAb: Hepatitis B core antibody
HBsAg: Hepatitis B surface antigen
HBV: Hepatitis B virus
HCV: Hepatitis C virus
HCV RNA: Hepatitis C virus ribonucelic acid
HIV: Human immunodeficiency virus
NICE: National Institute for Health and Care Excellence
PHE: Public Health England
TB: Tuberculosis
UK: United Kingdom
WHO: World Health Organisation
