# Supplementary material for: What do primary care staff know and do about blood borne virus testing and care for migrant patients? A national survey
Source: BMC Public Health. 2021 Feb 11;21:336. doi: 10.1186/s12889-020-10068-x (PMC7877334; doi:10.1186/s12889-020-10068-x)
Supplement: Supplementary file 1 — Additional file 1. Full online questionnaire. Text version of the online questionnaire used for the survey. [file 12889_2020_10068_MOESM1_ESM.pdf]

## Additional file 1: Full online questionnaire

# GP knowledge, attitudes, policy and practice regarding blood borne viruses (BBV) among migrants, including refugees and asylum seekers

Welcome

Public Health England is carrying out a survey of GPs' knowledge, attitudes, policy and practice on BBV testing and care among migrants including refugees and asylum seekers.

This is part of a larger programme of work to identify good practice and gaps in blood-borne virus service provision for these populations.

We would be grateful if you would complete this short survey.

**1. Please tell us who you are?**

**GP Partner** ☐
**Salaried GP** ☐
**Locum GP** ☐
**Practice Nurse** ☐
**Practice Manager** ☐
**Other, please specify**

## Section 1. Knowledge of migrant health, policy and recommendations

**2. To the best of your knowledge, as of August 2017 which of the following services are free to all irrespective of migration status?**

GP and nurse consultations in primary care Yes ☐ No ☐ NK ☐
Emergency departments and walk in centres Yes ☐ No ☐ NK ☐
Communicable disease services Yes ☐ No ☐ NK ☐
Sexually transmitted disease services Yes ☐ No ☐ NK ☐
Family Planning Services Yes ☐ No ☐ NK ☐
Treatment of physical or mental conditions caused by torture, female genital mutilation, domestic or sexual violence Yes ☐ No ☐ NK ☐
Operations or outpatient services in secondary care Yes ☐ No ☐ NK ☐
Hepatitis B and C testing, diagnosis and management Yes ☐ No ☐ NK ☐

**3. Public Health England has published a Migrant Health Guide online on GOV.UK.**

**Have you heard of the migrant health guide?** Yes ☐ No ☐
**If yes, have you used the guide?** Yes ☐ No ☐ NA ☐

**If you have not used the Migrant Health Guide, skip to Section 2**

**If you have used the Migrant Health Guide, did you use it and find it helpful when looking for information on?**

| | Used it and found it helpful | Used it but did not find it helpful for this | Did not use it |
|---|---|---|---|
| Communicable diseases | ☐ | ☐ | ☐ |
| Non-communicable health concerns | ☐ | ☐ | ☐ |
| NHS entitlements | ☐ | ☐ | ☐ |
| Country specific advice | ☐ | ☐ | ☐ |
| Assessing new patients from overseas | ☐ | ☐ | ☐ |
| Culture, spirituality and religion | ☐ | ☐ | ☐ |
| Language interpretation | ☐ | ☐ | ☐ |
| Travel to visit friends and relatives | ☒ | ☐ | ☐ |
| Human trafficking | ☐ | ☐ | ☐ |

## Section 2. Perception of migrant health care needs

**6. What do you think are the biggest challenges/ barriers that migrant populations face in accessing health care? Please give the top three, in your opinion.**

| | Asylum seekers | All migrants |
|---|---|---|
| **1** | | |
| **2** | | |
| **3** | | |

**7. How often do you see migrant patients?**

Daily ☐ Regularly (at least once a week) ☐ Rarely (less than once a week) ☐

**8. Which of the following issues do you consider when speaking to new migrant patients?**

| | All new migrants | Asylum seekers / refugees only | Never consider this | Situation specific |
|---|---|---|---|---|
| How long have they been in the UK | ☐ | ☐ | ☐ | ☐ |
| How much social support | ☐ | ☐ | ☐ | ☐ |

| | | | | |
|---|---|---|---|---|
| they have | | | | |
| Integration into UK society | ☐ | ☐ | ☐ | ☐ |
| Housing | ☐ | ☐ | ☐ | ☐ |
| Family situation | ☐ | ☐ | ☐ | ☐ |
| Migration history | ☐ | ☐ | ☐ | ☐ |
| Experiences of torture/violence | ☐ | ☐ | ☐ | ☐ |
| Disability/special needs | ☐ | ☐ | ☐ | ☐ |
| Diet/Nutrition | ☐ | ☐ | ☐ | ☐ |
| Vaccination history | ☐ | ☐ | ☐ | ☐ |
| Sexual health advice and screening | ☐ | ☐ | ☐ | ☐ |
| Family planning | ☐ | ☐ | ☐ | ☐ |
| TB screening | ☐ | ☐ | ☐ | ☐ |
| HIV risk assessment | ☐ | ☐ | ☐ | ☐ |
| Hep B risk assessment | ☐ | ☐ | ☐ | ☐ |
| Hep C risk assessment | ☐ | ☐ | ☐ | ☐ |
| Sickle cell/thalassaemia risk assessment | ☐ | ☐ | ☐ | ☐ |
| Psychological well-being | ☐ | ☐ | ☐ | ☐ |
| Dental health | ☐ | ☐ | ☐ | ☐ |
| Vision and hearing | ☐ | ☐ | ☐ | ☐ |

## Section 3. Patient information received for refugees and asylum seekers

**9. Do you receive patients from the following refugee resettlement programmes?**

Gateway Resettlement Programme Yes ☐ No ☐ NK ☐
Syrian Vulnerable Persons Resettlement Scheme Yes ☐ No ☐ NK ☐
Vulnerable Children's Resettlement Scheme Yes ☐ No ☐ NK ☐

**10. Do you receive patients who are new asylum seekers?** Yes ☐ No ☐ NK ☐

**11. If you receive patients who are part of refugee resettlement programmes, do you receive information from their pre-entry health assessment?**
Often/Always ☐ Sometimes ☐ Rarely ☐ Never ☐ Don't know ☐ NA ☐

**12.If you receive new asylum seekers, do you receive any clinical reports about health screening they have received in initial accommodation?**
Often/Always ☐ Sometimes ☐ Rarely ☐ Never ☐ Don't know ☐ NA ☐

**13. If yes, do you receive BBV testing results on patients who are asylum seekers?**
Often/Always ☐ Sometimes ☐ Rarely ☐ Never ☐ Don't know ☐ NA

## Section 4. Current Practice Policy for BBV testing

The following questions are about GP practice policy for BBV testing.

If you work in more than one practice, please think about the practice that you are most familiar with and respond for that practice.

**14. What is the current policy in your practice for BBV testing for NEW migrants? Please read the options carefully and tick the boxes which best describe your GP practice current policy, or leave blank if none apply.**

| | HIV | HBV | HCV |
|---|---|---|---|
| Offered universally / 'opt out' basis to all new migrants | ☐ | ☐ | ☐ |
| Offered to all new migrants on ad hoc basis, depending on individual risk factors | ☐ | ☐ | ☐ |
| Offered universally / 'opt out' basis to asylum seekers / refugees only | ☐ | ☐ | ☐ |
| Offered to asylum seekers / refugees only on ad hoc basis, depending on individual risk factors | ☐ | ☐ | ☐ |
| Don’t know | ☐ | ☐ | ☐ |

**15. What is the current policy in your practice for BBV testing offered to EXISTING patients who are migrants from higher prevalence countries?**

| | HIV | HBV | HCV |
|---|---|---|---|
| Offered universally / 'opt out' basis to all new migrants | ☐ | ☐ | ☐ |
| Offered to all new migrants on ad hoc basis, depending on individual risk factors | ☐ | ☐ | ☐ |
| Offered universally / 'opt out' basis to asylum seekers / refugees only | ☐ | ☐ | ☐ |

☐ ☐ ☐

Offered to asylum seekers / refugees only on ad hoc basis, depending on individual risk factors

☐ ☐ ☐

Don't know

**16. How are existing patients who are migrants identified for BBV testing at your practice?**

Periodic flagging of GP system by automated software Yes ☐ No ☐ NK ☐
Periodic flagging of GP system by manual audit Yes ☐ No ☐ NK ☐
Opportunistic during consultation Yes ☐ No ☐ NK ☐
Don't know Yes ☐ No ☐ NK ☐

**17. What do you think are the barriers for BBV testing of migrant patients attending your practice?**

**18. What tests are requested when you test for hepatitis B?**

HBsAg ☐ HBcAb ☐ Not known ☐ Other (specify)

**19. What tests are requested when you test for hepatitis C?**

Anti-HCV ☐ HCV RNA ☐ Not known ☐ Other (specify)

**20. What tests are requested when you test for HIV?**

Anti-HIV ☐ Not known ☐ Other (specify)

**21. Do you use dried blood spot testing (DBS)?**
Yes ☐ No ☐ NK ☐

**22. Who is most likely to perform BBV testing at your practice?**
GP ☐ Phlebotomist ☐ Practice nurse ☐ Don't know ☐
Other (specify)

**23. Which of the following would be an incentive or motivation to test for BBVs in migrants?**

Performance payment structure Yes ☐ No ☐ NK ☐
Local targets Yes ☐ No ☐ NK ☐

National goals Yes ☐ No ☐ NK ☐
CCG recommendations Yes ☐ No ☐ NK ☐
PHE recommendation Yes ☐ No ☐ NK ☐
NICE guidance Yes ☐ No ☐ NK ☐
NHSE recommendations Yes ☐ No ☐ NK ☐
CMO letter Yes ☐ No ☐ NK ☐
None of the above Yes ☐ No ☐ NK ☐

**24. The following questions are about people diagnosed with hepatitis B or C.**

Are all persons positive for hepatitis B referred to secondary care? Yes ☐ No ☐ NK ☐

Are all persons positive for hepatitis C referred to secondary care? Yes ☐ No ☐ NK ☐

If you make referrals to secondary care, do you receive information or check whether the patient has attended? Yes ☐ No ☐ NK ☐

Do you follow up patients that have not attended secondary care appointments? Yes ☐ No ☐

Are close contacts of HBV infected cases offered hepatitis B testing? Yes ☐ No ☐ NK ☐

Are close contacts of hepatitis B cases offered hepatitis B vaccination? Yes ☐ No ☐ NK ☐

**25. What interventions/services are in place at your practice to facilitate BBV testing and care of migrant patients?**

Specially designated clinic Yes ☐ No ☐ NK ☐
Designated GP within a practice Yes ☐ No ☐ NK ☐
Longer appointments at registration Yes ☐ No ☐ NK ☐
Specific projects to register migrants Yes ☐ No ☐ NK ☐
Outreach facilities Yes ☐ No ☐ NK ☐
Health support teams Yes ☐ No ☐ NK ☐
Incentive scheme for GPs Yes ☐ No ☐ NK ☐

**26. What do you think are the barriers for your patients with BBV accessing secondary care?**

## Section 5. Your practice information

## 27. Practice details

**Practice name:**
**Practice Postcode:**
**Location of general practice**

**28. Practice Characteristics:**
**Number of patients**
**Number of Staff**

**29. Please select all staff types you have in your practice.**
**Staff types:** GP Partner ☐ Salaried GP ☐ GP Registrar ☐ Practice Nurse ☐ Nurse Practitioner ☐ HCA ☐ Midwife ☐ Physiotherapist ☐ Counsellor ☐ Phlebotomist ☐ Podiatrist ☐ Other therapist ☐ Practice Manager ☐ Reception ☐ Administrator ☐ Medical Secretary ☐ Dispenser ☐ Other Staff ☐

**30. Can you give us an estimate of the proportion of your practice population that are:**
New migrants (<5 years in the UK)
Asylum seekers/refugees
Other migrants (please specify)

**31. What database system does your practice use?**

**EMIS** ☐
**SystmOne (TPP)** ☐
**Microtest** ☐
**Vision (INPS)** ☐
**Not known** ☐
**Other, please specify**

**32. Does your practice routinely record the following on practice systems?**

| | |
|---|---|
| Patient ethnicity | Yes ☐ No ☐ NK ☐ |
| Patient country of birth | Yes ☐ No ☐ NK ☐ |
| Hepatitis diagnosis | Yes ☐ No ☐ NK ☐ |
| Hepatitis prescription | Yes ☐ No ☐ NK ☐ |

**33. What do you think are the top 3 countries of birth of migrants registered with your practice?**
1.
2.
3.

**34. What do you think are the main reasons for migration for new migrants in your practice area?**

| | Main reason | Common reason | Not a common reason | NK |
|---|---|---|---|---|
| Economic (e.g. work) | ☐ | ☐ | ☐ | ☐ |
| Asylum (i.e. fleeing conflict or persecution) | ☐ | ☐ | ☐ | ☐ |
| Study | ☐ | ☐ | ☐ | ☐ |
| Accompanying/joining family | ☐ | ☐ | ☐ | ☐ |
| Other | ☐ | ☐ | ☐ | ☐ |

**35. These questions are about interpreter and outreach facilities offered by your practice.**

Do you offer interpreter facilities? Yes ☐ No ☐ NK ☐
Do you offer any outreach services? Yes ☐ No ☐ NK ☐
Do you offer any outreach services specifically for migrants/vulnerable migrants? Yes ☐ No ☐ NK ☐

**36. How could health and social care services be improved to meet the needs of recent migrants to your area?**

**37. Please name any refugee or migrant support or advocacy groups/organisations in your area that you would recommend to a migrant patient.**

**Survey completed**

**Thank you for taking the survey!**
