# Supplementary material for: What do primary care staff know and do about blood borne virus testing and care for migrant patients? A national survey
Source: BMC Public Health. 2021 Feb 11;21:336. doi: 10.1186/s12889-020-10068-x (PMC7877334; doi:10.1186/s12889-020-10068-x)
Supplement: Supplementary file 2 — Additional file 2. Shorter paper questionnaire. PDF of the paper questionnaire that was distributed at the RCGP and Best Practice in Primary Care conferences. [file 12889_2020_10068_MOESM2_ESM.pdf]

Public Health England

Protecting and improving the nation's health

GP knowledge, attitudes, policy and practice regarding blood borne viruses (BBV) among migrants, including refugees and asylum seekers

Public Health England (PHE) is carrying out a survey of GPs' knowledge, attitudes, policy and practice on BBV testing and care among migrants including refugees and asylum seekers. This is part of a larger programme of work to identify good practice and gaps in BBV service provision for these populations.

We would be grateful if you would complete this short survey.

Email address: ....

Please tell us who you are

GP Partner ☐ Salaried GP ☐ Locum GP ☐ Practice Nurse ☐ Practice Manager ☐

Other, please specify: ....

Where is your practice based?

East of England ☐ London ☐ Midlands ☐ North East ☐ North West ☐ South East ☐ South West ☐

1 To the best of your knowledge, as of August 2017 which of the following services are free to all irrespective of migration status in England?

GP and nurse consultations in primary care	Yes ☐	No ☐	Not known ☐
Emergency departments and walk in centres	Yes ☐	No ☐	Not known ☐
Communicable disease services	Yes ☐	No ☐	Not known ☐
Sexually transmitted disease services	Yes ☐	No ☐	Not known ☐
Family Planning Services	Yes ☐	No ☐	Not known ☐
Operations or outpatient services in secondary care	Yes ☐	No ☐	Not known ☐
Hepatitis B and C testing, diagnosis and management	Yes ☐	No ☐	Not known ☐
Treatment of physical or mental conditions caused by torture, female genital mutilation, domestic or sexual violence	Yes ☐	No ☐	Not known ☐

2 PHE publishes a Migrant Health Guide online (GOV.UK.); Have you heard it?

Yes ☐ No ☐

3 Do you see migrant patients in your practice?

Frequently ☐ Sometimes ☐ Rarely ☐

4 Which of the following issues would you consider when speaking to new migrant patients?

	All new migrants	Asylum seekers/ refugees only	Never consider this	Situation specific
Vaccination history	☐	☐	☐	☐
Sexual health advice and screening	☐	☐	☐	☐
Family planning	☐	☐	☐	☐
TB screening	☐	☐	☐	☐
HIV risk assessment	☐	☐	☐	☐
Hepatitis B (HBV) risk assessment	☐	☐	☐	☐
Hepatitis C (HCV) risk assessment	☐	☐	☐	☐

5 What is the current policy for BBV testing in the practice you predominantly work in:

New Migrants?

	HIV	HBV	HCV
Offered universally/'opt out' basis	☐	☐	☐
Offered on ad hoc basis, depending on individual risk factors	☐	☐	☐
None of the above	☐	☐	☐
Don't know	☐	☐	☐

EXISTING patients from high prevalence countries?

	HIV	HBV	HCV
Offered universally / 'opt out' basis	☐	☐	☐
Offered on ad hoc basis, depending on individual risk factors	☐	☐	☐
None of the above	☐	☐	☐
Don't know	☐	☐	☐

6 Which of the following would be an incentive or motivation to test for BBVs in migrants?

Performance payment structure	Yes ☐	No ☐	Not known ☐
Local targets	Yes ☐	No ☐	Not known ☐
National goals	Yes ☐	No ☐	Not known ☐
CCG recommendations	Yes ☐	No ☐	Not known ☐
PHE recommendation	Yes ☐	No ☐	Not known ☐
NICE guidance	Yes ☐	No ☐	Not known ☐
NHSE recommendations	Yes ☐	No ☐	Not known ☐
CMO letter	Yes ☐	No ☐	Not known ☐
None of the above	Yes ☐	No ☐	Not known ☐

Would you be happy for us to email a longer survey regarding BBVs among migrants?

Yes ☐ No ☐

Thank you for taking the survey!
