# Supplementary material for: What do primary care staff know and do about blood borne virus testing and care for migrant patients? A national survey
Source: BMC Public Health. 2021 Feb 11;21:336. doi: 10.1186/s12889-020-10068-x (PMC7877334; doi:10.1186/s12889-020-10068-x)
Supplement: Supplementary file 3 — Additional file 3. Definitions sheet which accompanied the questionnaire. [file 12889_2020_10068_MOESM3_ESM.pdf]

Definitions

People are often unclear about the definitions of terms used in relation to migration. To help with completing the survey, some definitions of terms used are given below.

Migrant

A migrant is someone who changes their country of usual residence, in whatever manner, for whatever reason and irrespective of their legal status. The UN defines a migrant as someone who moves to a country for a period of 12 or more months, effectively making it their new country of usual residence.

The majority of migrants to the UK choose to move to the UK for reasons such as finding work, education or joining family but a small proportion come because of fears for their safety in their home country. These are termed refugees or asylum seekers depending on their legal status within the UK.

Refugee

A refugee is someone who has fled their home country for reasons of feared persecution, conflict, generalised violence or other circumstances that have seriously disturbed public order and are seeking protection in another country.

In the UK, for a person to be officially recognised as a refugee they first have to make claim for asylum and have this accepted by the government.

Asylum seeker

An asylum seeker is a person who seeks safety from persecution or serious harm in a country other than their own and has formally applied for asylum (i.e. refugee status) from that country, but is awaiting a decision on their application.

Refused asylum seeker

A refused asylum seeker is someone who has had their claim for asylum refused and who is not waiting for a decision on any other claim for protection.

Undocumented migrant

While there is no universally accepted definition of an undocumented, or irregular, migrant, from the destination country's perspective, an undocumented migrant is someone who has entered, stayed or worked in a country without the necessary authorisation or documents required under immigration regulations.
