# Supplementary material for: What do primary care staff know and do about blood borne virus testing and care for migrant patients? A national survey
Source: BMC Public Health. 2021 Feb 11;21:336. doi: 10.1186/s12889-020-10068-x (PMC7877334; doi:10.1186/s12889-020-10068-x)
Supplement: Supplementary file 4 — Additional file 4. Example responses for barriers questions. Example responses for free text barriers questions, by theme and part of care pathway. [file 12889_2020_10068_MOESM4_ESM.pdf]

Example responses for barriers questions, by theme and part of care pathway

Theme	Health care	BBV testing	Secondary care
Language / culture	"language"<br>"language barrier"	"language"<br>"difficulties with language/interpretation taking too long"	"language barriers"<br>"language"<br>"costs of interpretation services"
Patient information / knowledge	"knowledge of what services available and how to access"<br>"uncertainty about eligibility"<br>"not knowing their rights"	"knowing what services available within GP practice"<br>"understanding about BBVs"<br>"awareness of treatment options"	"unfamiliarity with NHS system"<br>"lack of understanding about purpose of referral"<br>"understanding about service provision"
Staff information / knowledge	"uncertainty about entitlements"<br>"ignorance lack of training"	"realising not already tested"<br>"clinicians don't think to suggest BBV testing"<br>"HCP lack of knowledge"	"poor training and commitment of healthcare staff"<br>"lack of knowledge"<br>"ignorance of some secondary care staff to both their infectivity/perceived risk to staff and their access to healthcare"
Psychological	"fear"<br>"fear of deportation"<br>"fear of authority"	"fear of positive result"<br>"confidentiality concerns and fear of positive result and stigma"	"patients frightened of the outcome"<br>"stigma"<br>"fear"
Accommodation	"NFA"<br>"no fixed abode"<br>"frequently moved around"	"move around a lot"<br>"often transient"<br>"often have no fixed address or means of contact"	"moving from place to place"<br>"fixed address, move often"
Prejudice / discrimination	"discrimination"<br>"public perceptions"<br>"prejudice, hostile conditions"	"prejudice"	"fear of treatment by hospital staff due to immigration status"<br>"The Daily Mail and its racist propaganda"<br>"perceived hostility issues"
Financial (patient)	"cost"<br>"fear they will have to pay"<br>"money"		"cost"<br>"no money for transport to appointments"
Resource	"lack of capacity in system"<br>"appointment availability"<br>"cuts to services"	"resource implications and increasing demand on GP time and work overload"<br>"lack of time"<br>"time, multiple other issues being prevented"	"resource implications"<br>"lack of interpreters"<br>"time and incentive"
Geographical	"location"<br>"transport"		"distance from where they live and costs of transport"<br>"travel to hospital in city"
Service / organisational issues	"systems/bureaucracy"<br>"services not tailored for their needs"<br>"lack of specialist free services"	"lack of new patient assessments within our practice"<br>"no policy in place"<br>"poor IT system support"	"clinic arrangements/timetables"<br>"time delay between referral and appointment"<br>"lack of organised clinic. No structured approach"
